# Satellite DNAs in *Drosophila koepferae* (*repleta* group) reveal patterns of origin, chromosomal organization, transcription, and turnover in the *buzzatii* cluster

**DOI:** 10.1007/s10577-026-09814-5

**Published:** 2026-09-08

**Authors:** Ana Mattioli Laborne, John S. Heslop-Harrison, Gustavo C. S. Kuhn

**Affiliations:** 1 Departamento de Genética, Ecologia e Evolução, Instituto de Ciências Biológicas, Universidade Federal de Minas Gerais, Belo Horizonte, MG Brasil; 2 Department of Genetics and Genome Biology, University of Leicester, Institute for Environmental Futures, University of Leicester, Leicester, UK

**Keywords:** Satellite DNA, Repetitive DNA, Transposable elements, Heterochromatin, Centromeres

## Abstract

Satellite DNAs (satDNAs) are non-coding tandem repeats that can comprise more than 20% of eukaryotic genomes. They contribute to structural and regulatory processes in the genome and often evolve rapidly, shaping early stages of genetic differentiation between populations and species. Although *Drosophila* has long served as a model for studying satDNA biology, little is known about satDNAs in non-model *Drosophila* species, particularly within the *repleta* group, one of the most species-rich lineages in the genus. To reduce such bias, several studies have focused on the *buzzatii* cluster (*repleta* group). However, *D. koepferae* remained the only species lacking comprehensive satDNA data, limiting comparative analyses. Here, we used publicly available genomic sequencing data from two *D. koepferae* populations (Argentina and Bolivia) to characterize their satDNA content. Both populations share the same set of five satDNAs (CDSTR8, CDSTR138, CDSTR230, DBC-150 and CDSTR177), which together account for ~ 0,9% of the genomic DNA. We show that CDSTR177 originated through amplification of an internal segment of the *Galileo* transposable element, an event restricted to *D. koepferae*. All satDNAs localize to heterochromatic regions, with CDSTR138 most likely associated to the centromeres of most chromosomes. Transcripts from all satDNAs were detected, although at low levels. Our results provide new insights into the origin, genomic contribution, expression and evolution of satDNAs in the *buzzatii* cluster, support incipient differentiation between Argentinean and Bolivian populations of *D. koepferae* and contribute to clarifying the phylogenetic position of this species within the *buzzatii* cluster.

**Supplementary Information:**

The online version contains supplementary material available at https://doi.org/10.1007/s10577-026-09814-5.

## Introduction

Satellite DNAs (satDNAs) are a class of non-protein coding, tandemly repeated DNA sequences that frequently contribute to more than 20% of eukaryotic genomes (Walker 1971; Charlesworth et al. 1994; Plohl et al. 2012; Biscotti et al. 2015; Garrido-Ramos 2017). SatDNAs are predominantly found in large tandem arrays typically located in the heterochromatic regions of the chromosomes, notably centromeric, pericentromeric and subtelomeric regions (Tautz 1993; Charlesworth et al. 1986, 1994; Plohl et al. 2012). Although the repeat unit of most satDNAs falls within the 150–400 bp range, strikingly short monomers (e.g., 5–15 bp) are common in several groups, including many *Drosophila* species (Lohe et al. 1993; Wei et al. 2018; Silva et al 2023). However, shorter arrays or single repeats can be found dispersed throughout euchromatin (Kuhn et al. 2012; Brajković et al. 2012; Pita et al. 2017; Sproul et al. 2020a, b; Cabral-de-Mello et al. 2023; Grzan et al. 2023; Rico-Porras et al. 2024). Changes in heterochromatin content between chromosomes or species often correlates to changes in satDNA abundance (Kuhn et al. 2009; Plohl et al. 2012).

SatDNAs are among the fastest-evolving components of the eukaryotic genomes, with changes in both copy number and the primary sequence of monomers. In fact, several studies have shown that particular satDNA families are typically present in the genome of a single species or shared among closely related species (Ugarkovic and Plohl 2002). When a satDNA is shared between species, copies within a given species typically exhibit, on average, higher sequence identity than copies between species, a pattern known as concerted evolution (Dover 1982). A satDNA can also show drastic differences in abundance between species (Ugarkovic and Plohl 2002). In these contexts, satDNAs can provide useful markers for taxonomy, particularly for distinguishing cryptic species and for phylogenetic analyses, especially among recently diverged taxa (Biscotti et al. 2015; Garrido-Ramos 2017; Kuhn et al. 2021; Despot-Slade et al. 2022). In *Drosophila*, the rapid evolution of satDNAs has been linked to the origin of hybrid incompatibilities between species, suggesting that satDNAs may play a role in speciation (Ferree and Barbash 2009).

Once historically viewed as "junk DNA", mainly due to their non-protein coding nature, satDNAs are now recognized for their critical roles in a wide range of biological processes (Menon et al. 2014; Joshi and Meller 2017; Fingerhut et al. 2019; Mills et al. 2019; Jagannathan et al. 2018). Structurally, they contribute for the formation of heterochromatin (Dawe and Henikoff 2006; Melters et al. 2013) and occur at or near the centromeres in many eukaryotic species (Rošić et al. 2014). Beyond their structural importance for chromosomes and genomes, several studies have revealed that satDNAs transcripts are involved in a wide range of biological processes (Kuhn 2015; Shatskikh et al. 2020; Thakur et al. 2021; Šatović-Vukšić and Plohl 2023). In *Drosophila*, for example, satDNA transcripts participate in the assembly of functional centromeres (Rošić et al. 2014), regulation of gene expression (Lauria Sneidman and Meller 2021) and heterochromatin formation (Usakin et al. 2007). Unlike many eukaryotes that utilize DNA methylation for silencing repetitive elements, *Drosophila* largely lacks this system (Deshmukh et al. 2018). Instead, RNAi-dependent pathways and histone modifications are the primary mechanisms by which the chromatin states of satDNAs are established and maintained in the genus.

*Drosophila* species have been used as models to address several aspects of satDNA biology since the repeats were discovered in the early 1960 s (Beridze 1986; Dover 1982). Nevertheless, satDNA research in *Drosophila* remains focused in a few species, especially those from the *melanogaster* group (Kuhn et al. 2021). In particular, little is known about the satDNAs in the New World *repleta* group, which is one of the most numerous from the genus, including more than a hundred species (Oliveira et al. 2012; O’Grady and DeSalle 2018).

The *Drosophila buzzatii* cluster from the *repleta* group is a monophyletic group that contains seven closely related species: *D. buzzatii*, *D. koepferae*, *D. serido*, *D. antonietae*, *D. seriema*, *D. gouveai* and *D. borborema* (Manfrin and Sene 2006)*.* Like most species from the *repleta* group*,* they use cactus tissues as resources for breeding and feeding (Oliveira et al. 2012). All species are endemic to South America, except *D. buzzatii*, which although of South American origin, has dispersed to other continents through human-mediated transport of its cactus host plants (Fontdevila et al. 1982). As a consequence of their ecologically restricted niche, species of the *buzzatii* cluster are typically found in natural environments where their cactus host species occur, such as the Caatinga biome in northeast Brazil and the Chaco region in northern Argentina, southeastern Bolivia and western Paraguay, as well as in several smaller areas across central Brazil and the Atlantic coast (Manfrin and Sene 2006).

The *buzzatii* cluster comprises species with divergence times ranging from < 0.5 Mya to ~ 4 Mya (Manfrin and Sene 2006; Franco and Manfrin 2012; Santos et al. 2023), making it a valuable model for investigating early stages of satDNA evolution (Kuhn et al. 2021). The earliest event of cladogenesis within the cluster occurred around 4 Mya and separated the lineage leading to *D. buzzatii* from that leading to the remaining species. Accordingly, many morphological and genetic markers place *D. buzzatii* in a basal phylogenetic position within the cluster. The remaining species, have divergence times from < 0.5 Mya to ~ 2 Mya and are placed, except for *D. koepferae* (see below) in the so called “*serido* sibling set”, composed by *D. serido*, *D. antonietae*, *D. seriema*, *D. gouveai* and *D. borborema* (Manfrin et al. 2001; Manfrin and Sene 2006; Franco et al. 2010; Moreyra et al. 2019).

All seven species from the *buzzatii* cluster share a similar karyotype, consisting of four pairs of acrocentric autosomes, one pair of microchromosomes (or chromosome 6), and one pair of sex chromosomes (X and Y) (Baimai et al. 1983; Kuhn et al. 2007). Heterochromatin is largely restricted to the (peri)centromeric regions of the four pairs of acrocentric autosomes, covers most of the microchromosomes and Y chromosome, and is located in the pericentromeric region of the X chromosome. The karyotype of most species can be distinguished by the amount of heterochromatin present in the microchromosomes and sex chromosomes (Baimai et al. 1983; Kuhn et al. 2007).

In the last 10 years, studies based on sequenced genomes have enabled the characterization of the full satDNA repertoire in many representative species of the *buzzatii* cluster. These studies also showed marked differences in satDNA abundance, location and nucleotide sequences between species, suggesting extensive satDNA turnover (Kuhn et al. 2021). So far, six satDNAs have been identified in species from the *buzzatii* cluster: pBuM, that can be further divided in pBuM-1 (*alpha* ~ 180 bp long repeats) and pBuM-2 (*alpha/beta* ~ 370 bp long repeats) (Kuhn et al. 2008); DBC-150 (~ 150 bp long repeats) (Kuhn et al. 2007); CDSTR138 (~ 138 bp long repeats) (Lima et al. 2017; Laborne et al. 2024); CDSTR230 (~ 230 bp long repeats) (Laborne et al. 2024) and the CDSTR8 (~ 8 bp repeats, but also showing higher-order repeats containing up to 5 monomers) (Rossi et al. 2025). The pBuM was the only satDNA identified in *D. buzzatii*. The species *D. antonietae*, *D. serido*, *D. seriema*, *D. gouveai* and *D. borborema* share four satDNAs, pBuM, DBC-150, CDSTR138 and CDSTR230, while another satDNA, CDSTR8, is only present in *D. gouveai* and *D. borborema*. FISH experiments revealed that all satDNAs are located in heterochromatic regions. pBuM is the main centromeric component of *D. buzzatii* (Kuhn et al. 2008). In *D. serido*, *D. antonietae* and *D. seriema,* CDSTR138 is the main centromeric component. In *D. gouveai*, CDSTR138 and CDSTR8 are the main centromeric components (Rossi et al. 2025). The CDSTR230 was found in the heterochromatic regions (including centromeric) of the X and/or Y chromosomes and, in some cases, in the microchromosomes (Laborne et al. 2024; Rossi et al. 2025). The DBC-150 was found restricted to the microchromosomes (Kuhn et al. 2007).

*Drosophila koepferae,* which is endemic to the Chaco region, is the only species from the *buzzatii* cluster where satDNAs have not been studied using sequenced genomes. Studies using earlier methodological approaches, such as restriction enzyme digestion of genomic DNA and PCR-based assays, enabled identification of only one satDNA in its genome, DBC-150. In *D. koepferae*, DBC-150 was limited to the microchromosomes, as found in other species of the cluster (Kuhn et al. 2007). Therefore, the overall composition of satDNAs, and consequently of heterochromatin (including centromeric) in *D. koepferae* remains largely unknown. Interestingly, the pBuM family (pBuM-1 and pBuM-2 variants), which is present in high-copy number in most species of the *buzzatii* cluster, was not detected in *D. koepferae* based on DNA-DNA hybridization experiments, revealing that this species underwent a particularly strong event of satDNA turnover in its genome (Kuhn et al. 2021).

The satDNA knowledge gap in *D. koepferae* is also particularly critical because the phylogenetic position of *D. koepferae* within the *buzzatii* cluster is still unresolved (Manfrin and Sene 2006; Hurtado et al. 2019; Moreyra et al. 2019). While mitochondrial DNA markers suggest a sister relationship with *D. buzzatii* (Manfrin and Sene 2006; Franco et al. 2010; Moreyra et al. 2019), nuclear DNA markers support an alternative, incongruent or discordant topology, placing *D. koepferae* closer to the other species in the ‘*serido* sibling set’ (Hurtado et al. 2019).

Previous studies based on chromosomal inversions and allozyme markers (Fontdevila et al. 1988), and more recently on mitochondrial genomes (Moreyra et al. 2019) and nuclear genomic data (Moreyra et al. 2023) revealed incipient differentiation between Argentinean and Bolivian populations of *D. koepferae*. These differences suggest limited gene flow between populations and raise the question of whether differentiation between these two populations can also be detected at the satDNA level.

In this study, we present the first description of the satDNA landscape in *D. koepferae*, leveraging publicly available genomic sequencing data from two distinct populations (Argentina and Bolivia). We also used publicly available RNA-seq data to access satDNA transcription. By characterizing the full repertoire of satDNAs in *D. koepferae*, including their transcription activity, chromosome location and sequence evolution, we aim to fill a crucial gap in our understanding of satDNA biology within the *buzzatii* cluster. Furthermore, this study enabled us to investigate potential differentiation between Argentinean and Bolivian populations at the satDNA level, and to gain new insights into the still debated phylogenetic position of *D. koepferae* within the *buzzatii* cluster.

## Materials and methods

### Samples and genomic data

We used publicly available (NCBI) raw Illumina reads from two sequenced genomes of *Drosophila koepferae*: strain Ko7.1 from Argentina (SRX12630073; De Panis et al. 2022) and strain Ko11 from Bolivia (SRX18356637; Moreyra et al. 2023), hereafter referred to as DkoeA and DkoeB, respectively. The corresponding raw PacBio (RSII and Sequel) reads and assembled genomes (SRR22206171 and GCA_030914325.1 for DkoeA; SRR22206170 and GCA_030914345.1 for DkoeB) were also analyzed. For satDNAs transcriptional analysis, we used raw RNAseq reads from *D. koepferae* reared on *Trichocereus trerscheckii* (‘native’ condition; PRJNA858943; De Panis et al. 2022).

### Repetitive DNA analysis

The de novo identification of repetitive elements was performed using the RepeatExplorer2 pipeline on the Galaxy platform, including the TAREAN (Tandem Repeat Analyzer) module for satDNA detection (Novák et al. 2017, 2020) (Fig. S1). Quality control involved trimming reads to approximately 100 bp, initially applying Phred quality cutoff of both 10 and 20, with the requirement that 95% of bases exceeded the selected threshold. As both filtering strategies yielded highly similar clustering results, and because RepeatExplorer2 was used solely for identification and generation of satDNA consensus sequences, we selected the results obtained with default pipeline setting (Phred > 10) for further analysis. More restrictive quality cutoffs were avoided, as stringent filtering can lead to the selective depletion of certain repetitive sequences, particularly in the case of GC-rich repeats. Three replicate runs were conducted separately for each sequenced genome, followed by a comparative run combining reads from both populations. The following RepeatExplorer2 settings were applied: pair-end reads: yes; read sampling: no; select taxon and protein domain database version (REXdb): Metazoa version 3.0; advanced options: no; select queue: long. The resulting HTML reports were downloaded and manually examined. Clusters identified as organellar or contaminant DNA were excluded, and genomic proportions were recalculated after manual curation. BLASTn searches against the RepBase and GenBank databases were conducted to search for potential homology with annotated genetic elements.

### Satellite DNA identification

RepeatExplorer2 analyzed 5,904,235 from a total of 13,773,762 reads for DkoeA and 6,394,359 from a total of 69,558,804 reads for DkoeB. This analysis resulted in the identification of 201 and 209 repeat clusters for DkoeA and DkoeB, respectively (Table S1 and S2). The TAREAN pipeline classified the clusters as putative satDNAs and assigned them to two categories: High Confidence and Low Confidence. From the complete set of putative satDNAs identified by TAREAN, we selected for detailed analysis only those clusters containing a minimum of 500 copies in at least one of the analyzed genomes and showing no significant homology (> 70% identity over > 70% of sequence) to other genetic elements (as determined by BLASTn searches against the GenBank and RepBase databases) (Fig. S1). Clusters displaying partial homology limited to a small segment of a larger element were further inspected manually. For each selected cluster, full-length satDNA copies were manually extracted from the contigs assembled by RepeatExplorer and then manually curated in Geneious Prime, retaining only those with ≥ 70% identity to the consensus sequence generated by TAREAN.

To characterize the genomic organization of CDSTR177 and investigate its association with the *Galileo* transposable element, we performed BLASTn searches of the CDSTR177 consensus sequence against the assembled genomes of *D. koepferae* (DkoeA: GCA_030914325.1; DkoeB: GCA_030914345.1). CDSTR177 tandem arrays were identified by manual inspections using Geneious Prime 2025. We also performed BLASTn searches in the same assembled genomes to investigate the possible presence of the pBuM satellite DNA in *D. koepferae* genome.

### SatDNA evolution

The full-length satDNA copies extracted from RepeatExplorer contigs were aligned using MUSCLE (Edgar 2004) and evolutionary analyses were performed using the Maximum Likelihood (ML) method implemented in MEGA10 Software (Tamura 2021) (Fig. S1), with 1,000 bootstraps replicates. The best-fit evolutionary models were predicted for each nucleotide alignment. Trees were edited using TreeGraph 2 and FigTree v1.4.4 (Stöver and Müller 2010; available at http://tree.bio.ed.ac.uk/software/figtree/).

### Probes for in situ hybridizations

Total genomic DNA was extracted (Fig. S1) from a pool of 20–30 adults of *D. koepferae* (strain B26-D2 from Famatina, Argentina; Baimai et al. 1983) with the Wizard Genomic DNA Purification Kit (Promega).

Satellite DNAs were amplified by PCR consisting of initial denaturation step at 95ºC for 5 min, followed by 30 cycles at 95ºC for 1 min, 47–55ºC for 1 min (based on the melting temperature of each primer) and at 72ºC for 1 min and final extension for 10 min at 72ºC. For the amplification of CDSTR230, we used primers CDSTR230F (GTCAGAATGTTGGAGAGC) and CDSTR230R (CATCCGCCAATATCTTATACA); and for CDSTR177, we used CDSTR177F (GATGAGAAGTTTTAAGCG) and CDSTR177R (CAGTTTTGGTATAACGTTC).

Products were purified using Wizard SV Gel and PCR Clean-Up System (Promega) and sequenced by the Sanger method. The probes used for FISH were labeled with digoxigenin 11-dUTP or biotin 11-dUTP by nick translation with the DIG-nick and Biotin-nick translation mix (Roche Applied Science), respectively, except of the CDSTR54 probe, which was manufactured as a 5'-biotin-labeled probe (Síntese Biotecnologia LTDA) containing one monomer of 54 bp.

Probes for the CDSTR138, CDSTR198 satDNAs were prepared as described above using primers designed by Laborne et al. (2024). For the CDSTR8, we used a 5′-biotin-labeled oligonucleotide probe synthesized directly by Síntese Biotecnologia LTDA, consisting of 40 bp corresponding to the consensus repeat derived from TAREAN, containing two of the three variants (as detailed in Rossi et al. 2025). The direct synthesis approach was chosen because the short repeat unit allowed the entire probe to be obtained as a single synthetic fragment, avoiding the need for PCR amplification and nick translation.

### Fluorescent in situ hybridization (FISH)

Mitotic and polytene chromosomes were obtained from neuroblasts and salivary glands, respectively, of third instar larvae of *D. koepferae* (strain B26-D2), according to methodology described in Baimai (1977) and Ashburner (1989), with modifications. For metaphase chromosomes preparation, we incubated the neuroblasts in Colcemid for 12 min and in the 0.7% trisodium-citrate solution for 15–17 min.

Probe detection was performed using anti-digoxigenin-Rhodamine and avidin-FITC (Roche Applied Science). Chromosome hybridizations (Fig. S1) were conducted as described by Dias et al. (2014) with minor modifications, including a 1 min and 15 s treatment with 0.07 M NaOH. Chromosomes were counterstained with DAPI (4,6-diamidino-2phenylindole) in antifade reagent (SlowFade, Invitrogen) and analyzed under an Axio Imager A2 epifluorescence microscope equipped with the AxiocamMRm camera (Zeiss). Images were captured with Axiovision (Zeiss).

### Transcriptional analysis

Total RNA-seq data from a pool of 15 mixed sex third-instar larvae randomly sampled from three different genotypes of *D. koepferae* from Northwestern Argentina was obtained from a study conducted by De Panis et al. (2022) (‘native’ condition sample; PRJNA858943). The RNA data were obtained from three genotypes that differ in chromosomal inversion arrangements, thus representing biological replicates (De Panis et al. 2022). Raw reads were processed to remove adapters and low-quality reads using Trimmomatic (Galaxy Version 0.39 + galaxy2). The filtered reads were then mapped against selected repetitive DNA consensus sequences using Geneious Prime. Expression levels were normalized using the FPKM (fragments per kilobase per million mapped reads) method.

## Results

### General repetitive landscape

**Fig. 1:**
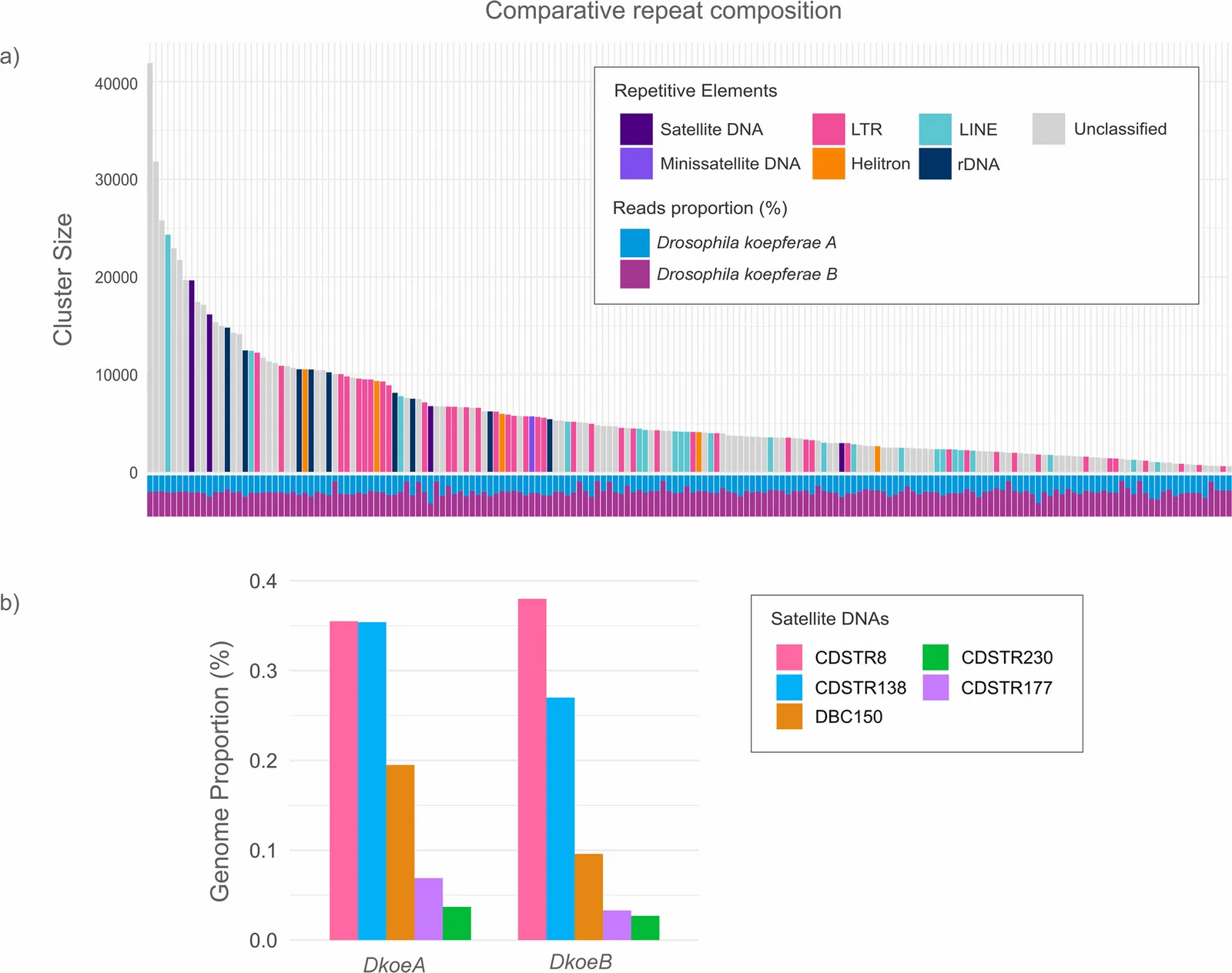
**a** Bar plot of the repeat class composition for the comparative cluster analysis reported by RepeatExplorer2 from the two *D. koepferae* populations (DkoeA and DkoeB). **b** Genome proportion of satellite DNAs identified in the two populations of *D. koepferae*

Comparative analysis of RepeatExplorer2 clusters (Tables S1 and S2; Fig. S2) indicated that the two *D. koepferae* populations (DkoeA and DkoeB) exhibit very similar repetitive DNA landscapes, despite some minor quantitative variations (Fig. 1a; Fig. S3). In both populations, repetitive DNAs accounted for ~ 19% of the genome. Among the clusters that were successfully classified by the automated pipeline, transposable elements (TEs) are the most abundant class of repetitive elements, with long terminal repeat (LTR) elements predominating (3.9–4.2%), followed by LINEs (non-LTR retrotransposons; 1.8–2.0%) and Helitrons (DNA transposable elements; ~ 0.5%). Satellite DNAs contributed only 0.8–0.9% of the genome (see below). However, a substantial fraction of the non-satellite repeat clusters remained unclassified by the automated approach (Fig. 1a; Fig. S3), most likely representing divergent and/or fragmented TEs.

### SatDNA characterization and inter-population analysis

After manual curation of TAREAN results (Tables S3 and S4), five satDNA families met our classification criteria and were therefore retained for subsequent analysis (Table 1; ESM3). Four of these families were previously described in other *buzzatii* cluster species*:* CDSTR8 (Rossi et al. 2025), CDSTR138 (Lima et al. 2017), DBC-150 (Kuhn et al. 2007) and CDSTR230 (Laborne et al. 2024). One family (CDSTR177) is described here for the first time. Both populations showed similar relative contributions for each satDNA, although some quantitative differences were detected (Fig. 1b).

**Table 1:** General features of the five satDNA identified by TAREAN in *D. koepferae*

	Satellite DNA	Genome Proportion (%)	Consensus Length (bp)	AT content (%)	Satellite Probability (%)	Copy Number Estimation
*D. koepferae (A)*	CDSTR8	0.355	40	70	0.994	15,975
	CDSTR138	0.354	138	70.3	0.989	4,617
	DBC-150	0.195	150	42	0.979	2,340
	CDSTR230	0.069	261	72.8	0.976	476
	CDSTR177	0.036	177	65	0.0651	366
*D. koepferae (B)*	CDSTR8	0.383	40	70	0.986	17,235
	CDSTR138	0.279	138	70.3	0.989	3,639
	DBC-150	0.098	150	42.7	0.979	1,176
	CDSTR230	0.034	261	72.8	0.976	234
	CDSTR177	0.027	177	65	0.0485	275

**Fig. 2:**
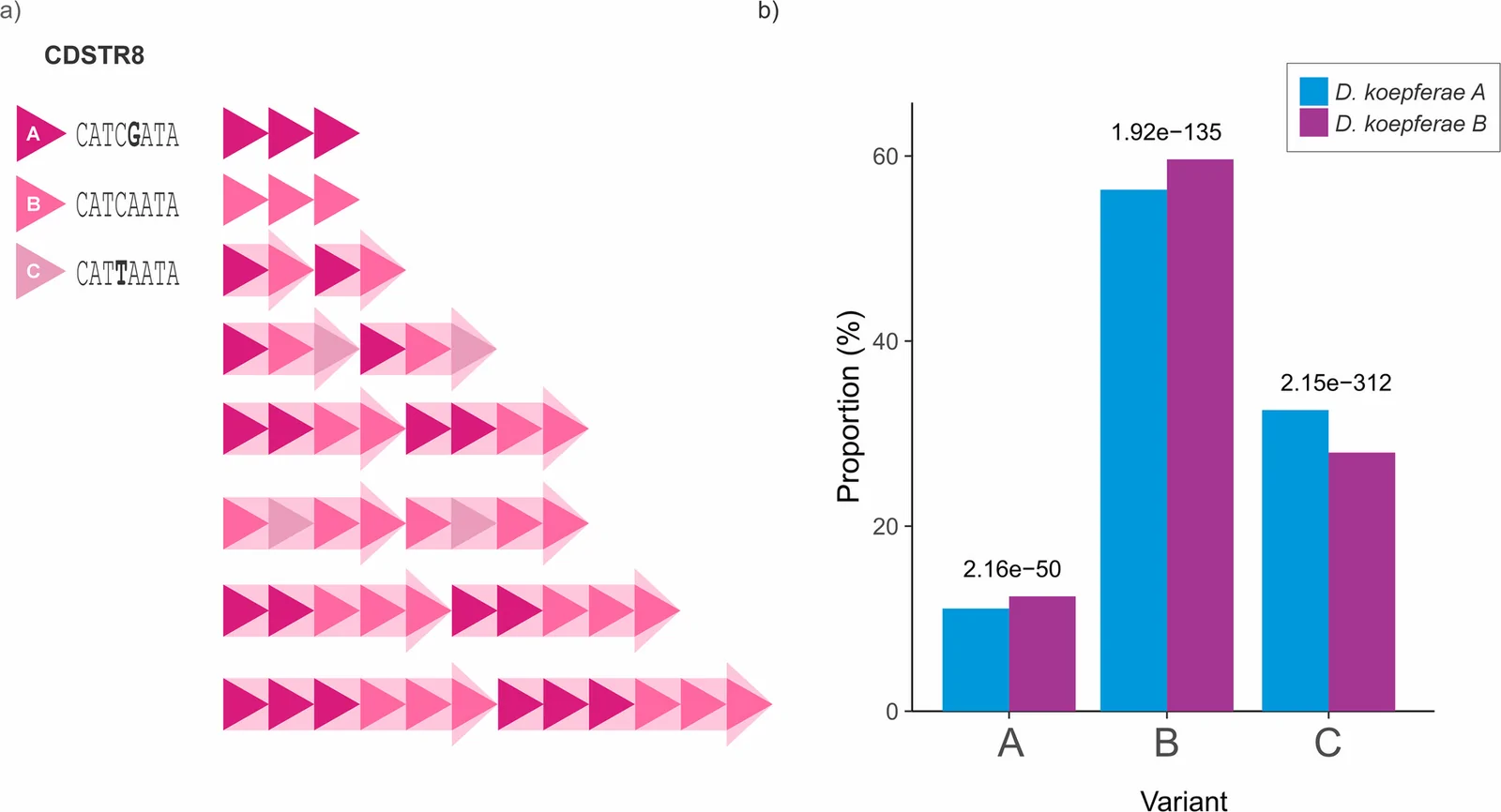
**a** Monomer variants and Higher-Order Repeat (HOR) structure of CDSTR8 found in the contigs retrieved by RepeatExplorer2. **b** Proportion of the three variants in two populations of *D. koepferae*. Bars show mean proportion in raw reads; p-values (Bonferroni corrected) from two-proportion Z-tests are indicated above each variant

The AT-rich (~ 70%) CDSTR8 was the most abundant satDNA in both *D. koepferae* populations, accounting for ~ 0.35% of the genomic DNA (Table 1; Fig. 1b). Although TAREAN returned a 40 bp consensus for both populations (Table 1), manual inspection of contigs from RepeatExplorer2 clusters showed that CDSTR8 is composed of three major 8 bp repeat variants (A: CATCGATA; B: CATCAATA; or C: CATTAATA), which differ from one another by a single nucleotide substitution. These variants occur as simple monomers (8 bp tandem repeats) or in the form of multimeric higher-order repeats (2-mer to 6-mer repeats) (Fig. 2a). Because the short repeat length precluded robust maximum-likelihood inference, we used variants A, B and C as queries in 100%-identity searches against the PacBio (RSII and Sequel) raw reads, and counted exact matches for each variant, considering only reads containing more than five copies. These data were generated on PacBio RSII and Sequel platforms, which produce continuous long reads (CLRs) with a relatively high error rate (~ 10–15%). Consequently, sequence errors may both erase true variants matches (leading to underestimation) and create spurious exact matches to another variant (leading to overestimation). The requirement of at least five copies per read mitigates the impact of random errors, but absolute variant counts should be interpreted with caution. The observed differences in relative abundance of the three variants are statistically significant (Z-test; Fig. 2b; Table S5), yet these results should also be viewed with caution given the limitations of CLR data explained above.

**Fig. 3:**
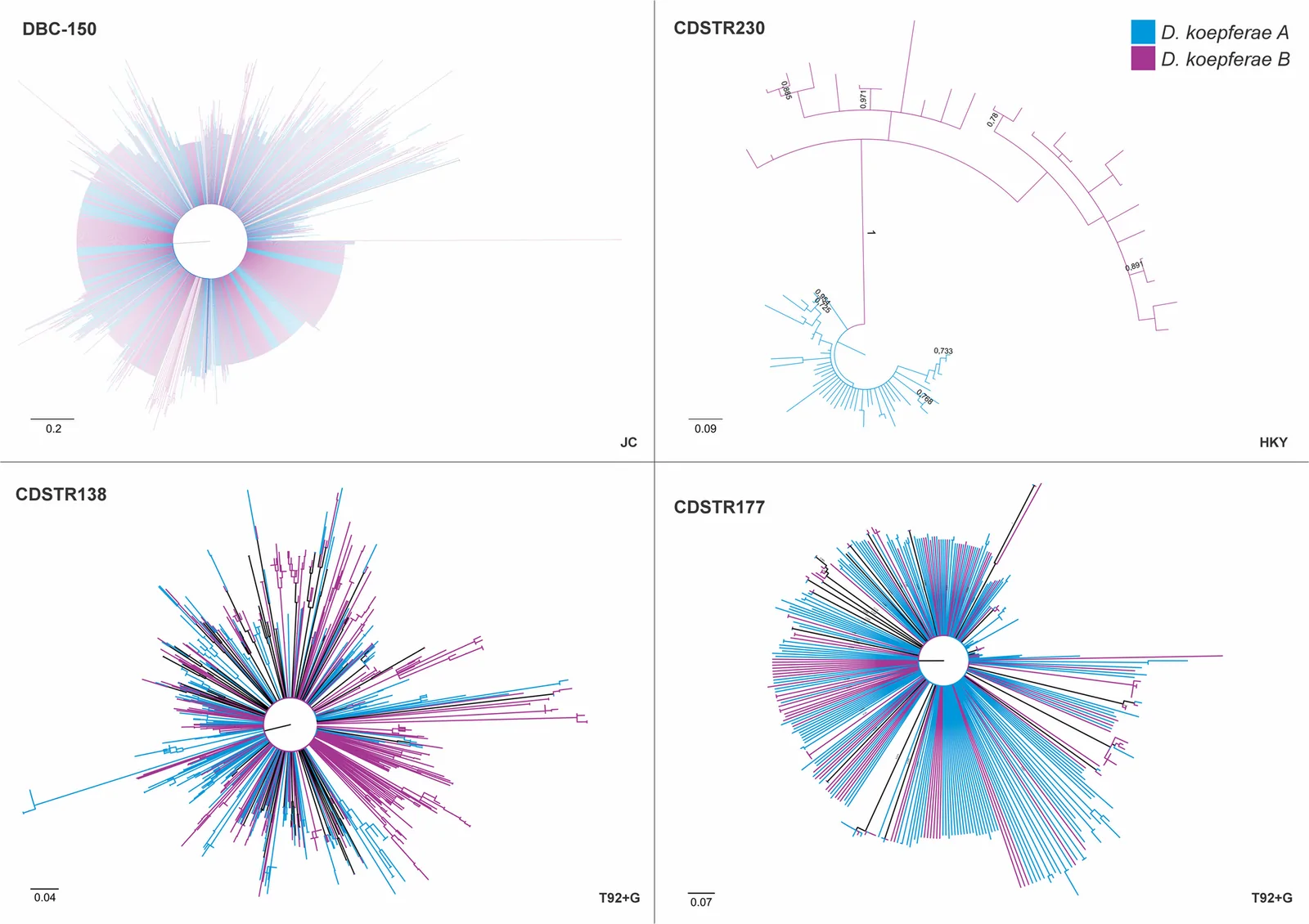
Maximum-likelihood trees of repeats from four selected (clusters containing ≥ 500 copies in at least one of the analyzed genomes and showing no similarity to other genetic elements) satDNA families in *D. koepferae*. SatDNAs repeats are color-labeled according to their population of origin (**A** and **B**). Best-fit substitution models were inferred for each tree. Nodes with bootstrap support below 20% were collapsed, and only values above 70% are shown

The CDSTR138 AT-rich (~ 70%) satDNA was also highly present, tying CDSTR8 as the most abundant satDNA in DkoeA (0.35 genomic proportion), and accounting for ~ 0.28% of the genome in DkoeB (Table 1; Fig. 1b). In both populations, TAREAN returned a 138 bp consensus (Table 1; Fig. S4). A ML tree (Fig. 3) showed branches containing extensive intermixing of repeats from both populations, suggesting no evidence for inter-population differentiation at the nucleotide level for this satDNA.

The DBC-150 (~ 58% GC) satDNA was also abundant, contributing 0.195% of the genomic DNA in DkoeA and 0.098% in DkoeB (Table 1; Fig. 1b). TAREAN returned a 150 bp consensus sequence for both populations (Table 1; Fig. S5). As observed for CDSTR138, a ML tree (Fig. 3) did not reveal inter-population differentiation for this satDNA at the nucleotide level.

The AT-rich (~ 73%) CDSTR230 satDNA accounted for 0.07% of the genomic DNA in DkoeA and 0.035% in DkoeB (Table 1; Fig. 1b). TAREAN returned a 261 bp consensus sequence for both populations (Table 1; Fig. S6). In contrast to CDSTR138 and DBC-150, a ML tree (Fig. 3) with CDSTR230 repeats showed strong inter-populational differentiation, with repeats from each population clustering into two main population-specific branches supported by bootstrap values > 70%.

**Fig. 4:**
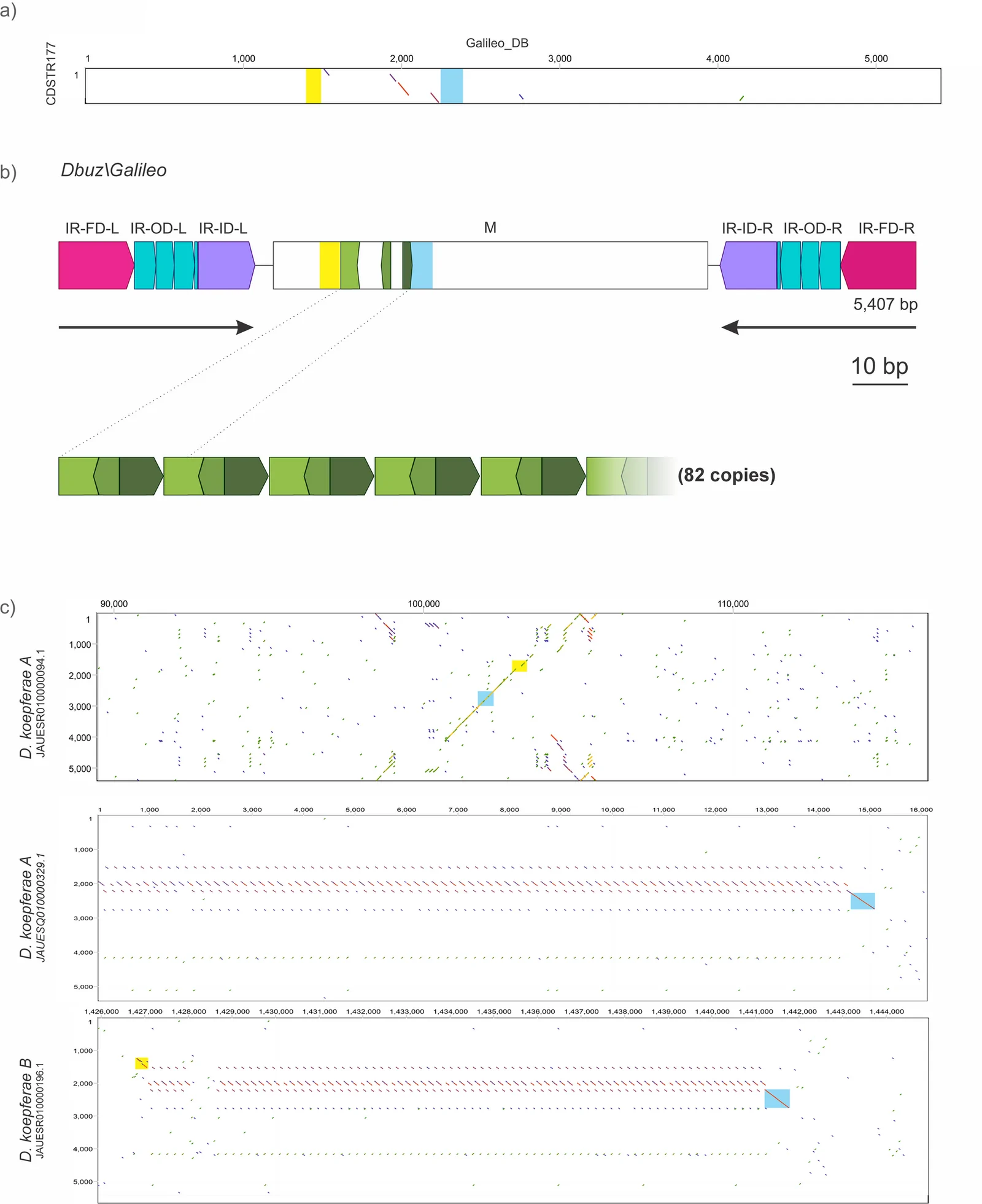
**a** Dot-plot showing homology between CDSTR177 and *Galileo* transposon. **b** General representation of the *Galileo* transposon (top) with the inverted-repeat architecture (IR-FD, IR-OD, IR-ID) flanking a central “M” region. The lower panel displays a tandem array of up to 82 copies of the internal repeat CDSTR177 found in the assembled genomes of *D. koepferae* (populations A and B). **c** Dot plots showing three contigs from the *D. koepferae* assembled genomes containing copies of the CDSTR177 and their flanking region matching the corresponding region of the *Galileo* transposon (indicated by the yellow and blue shades)

The AT-rich (~ 65%) CDSTR177 was the only satDNA detected in *D. koepferae* that has not been previously described. This family contributed 0.036% of the DkoeA genome and 0.027% of the DkoeB genome (Table 1; Fig. 1b). TAREAN returned a 177 bp consensus sequence for both populations (Table 1; Fig. S7). Manual inspection of the genome assemblies identified 221 full-length monomers in DkoeA distributed in five arrays (range: 4–82 copies) (Fig. 4a) and 139 monomers in DkoeB also in five arrays (range: 2–71 copies). The lower copy number detected in the assemblies compared to the copy number estimation calculated using TAREAN data (Table 1) likely reflects the limited contiguity of the assemblies (N50 = 2.3 Mb for DkoeA and 16.5 Mb for DkoeB). Despite being derived from PacBio long reads, the low N50 indicates that repeat-rich regions were not fully resolved by the assembler, leading to collapsed or fragmented representation of this satDNA. BLASTn similarity searches against GenBank and Repbase databases revealed homology to the central region of *Galileo*, a transposable element from the *P* superfamily, with 80% of query cover and 78% of identity (Marzo et al. 2008) (Fig. 4a, b). All CDSTR177 copies identified, including partial matches (~ 60% identity), were consistently flanked by *Galileo* sequences (Fig. 4c). Searches using *Galileo* sequence from *D. buzzatii* as query recovered a subset of copies falling into three categories: (i) copies containing only the terminal inverted repeats (TIRs), and therefore uninformative with respect to CDSTR177; (ii) copies containing a single CDSTR177 monomer (Fig. 4c; top); and (iii) copies containing multiple CDSTR177 monomers organized in tandem (Fig. 4c; middle and bottom). Notably, both single-copy and tandem-array insertions were flanked by the same *Galileo* region (Fig. 4c). A ML tree (Fig. 3) showed extensive intermixing of CDSTR177 repeats from both populations, suggesting no detectable inter-population differentiation at the nucleotide level.

Given that pBuM satellite family was not recovered as a repetitive cluster by RepeatExplorer2/TAREAN in either *D. koepferae* populations, we further searched for this satDNA directly in the genome assemblies using BLASTn, with consensus sequences of pBuM-1 from *D. buzzatii* (GenBank accessions AY520881.1 and EF620847.1) and pBuM-1 and pBuM-2 from *D. serido* (Laborne et al. 2024) as queries. This search identified only a small number of isolated pBuM copies in both DkoeA and DkoeB assemblies, with no evidence of tandem organization. The most complete copies identified in each population are shown in Fig. S8 (DkoeA) and Fig. S9 (DkoeB).

### Other tandem repeats

**Fig. 5:**
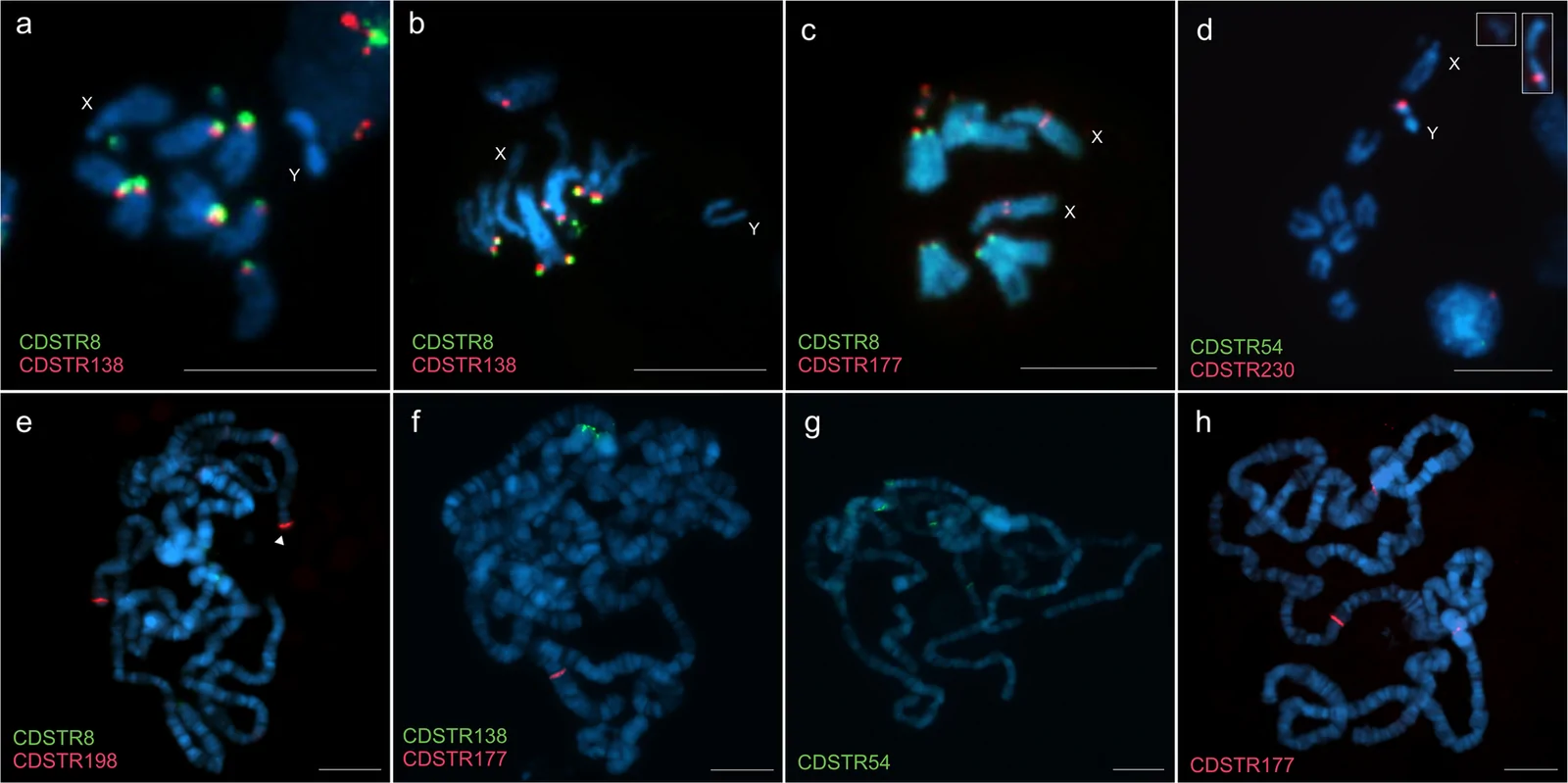
Fluorescent in situ hybridization in *D. koepferae* chromosomes. **a, b, c, d** mitotic chromosomes; **e, f, g** polytene chromosomes. White arrow points to telomeric signal. Probes are indicated in red or green. Scale bars represent 5 µM (up), and 10 µM (down)

In addition to the satDNAs described above, RepeatExplorer2/TAREAN also detected additional but minor (low abundance) tandem-repeat families in *D. koepferae*. The CDSTR198 had a consensus sequence of 188 bp. This family has also been reported in other species from the *buzzatii* cluster, but as a minisatellite DNA (Lima et al. 2017; Laborne et al. 2024). FISH with a CDSTR198 probe on mitotic chromosomes of *D. koepferae* yielded no detectable signals (data not shown). In contrast, FISH on polytene chromosomes revealed CDSTR198 dispersed signals across euchromatic and telomeric regions (Fig. 5e). This result is consistent with a minisatellite DNA classification (Fig. 1a). Accordingly, CDSTR198 was excluded from satDNA-focused analysis.

RepeatExplorer2/TAREAN also identified a low-confidence putative satDNA that we named CDSTR54 (54 bp repeats). BLASTn searches yielded no significant similarity to annotated sequences either in the RepBase or Genbank databases. Inspection of the assembled genomes of both *D. koepferae* populations revealed CDSTR54 arrays of up to 218 copies, along with smaller arrays. FISH on mitotic chromosomes with a CDSTR54 probe showed no detectable signals, whereas FISH on polytene chromosomes revealed dispersed euchromatic signals (Fig. 5d, g). These results are more consistent with a minisatellite DNA. Accordingly, this family was excluded from subsequent analysis.

Manual inspections of additional tandem repeats retrieved by the TAREAN revealed other several clusters (e.g., Cl_185, Cl_187 and Cl_111 in DkoeA and Cl_200, Cl_164 and Cl_184 in DkoeB) with no significant BLASTn hits in RepBase or Genbank. However, given their occurrence in short arrays (up to 49 copies), they were interpreted as low-copy tandem repeats rather than bona fide satDNAs.

The ribosomal DNA spacers IGS-109TR and 225TR (Laborne et al. 2024; Stage and Eickbush 2007) were also identified by TAREAN as low-confidence satDNAs (clusters 13 and 11 in DkoeA and clusters 15 and 37 in DkoeB, respectively) (Table S3 and S4). Although these tandem repeats are derived from the rDNA genes and thus do not represent satDNAs, they were retained for transcriptional analysis given their genomic context within transcribed spacer region (see topic below on satDNA transcription).

### SatDNA chromosome location

The *D. koepferae* karyotype comprises four pairs of acrocentric autosomes, one pair of microchromosomes, and one pair of sex chromosomes (X and Y) (Baimai et al. 1983; Kuhn et al. 2007). The X chromosome is acrocentric and the Y is metacentric. Heterochromatin is largely restricted to the (peri)centromeric regions of the four pairs of acrocentric autosomes, covers most of the microchromosomes and Y chromosome, and forms a large pericentromeric block on the proximal region of the X chromosome.

To investigate the chromosome location of the satDNAs identified in this study, we performed FISH with satDNA probes on both mitotic and polytene chromosomes of *D. koepferae*. Mitotic chromosomes enable the visualization of satDNA signals in heterochromatic regions, where these sequences are typically enriched. In contrast, the highly endo-replicated polytene chromosomes allow detection of satDNA arrays located in the euchromatic regions (if any) but are generally not informative for heterochromatic regions as they remain under-replicated. In this context, heterochromatic centromeric regions coalesce into the chromocenter, where pericentromeric regions from different chromosomes remain under-replicated. The Y chromosome, being almost entirely heterochromatic, is also under-replicated in polytene chromosomes and therefore contributes to the formation of the chromocenter rather than forming distinct polytene bands.

On mitotic chromosomes, CDSTR8 mapped to the pericentromeric regions of all four pairs of acrocentric autosomes and the microchromosome pair (Fig. 5a, b). In polytene chromosomes, CDSTR8 signals were detected within the chromocenter, a finding consistent with its mapping to the entirely heterochromatic short arms of autosomes (Fig. 5e). The CDSTR138 also mapped to the pericentromeric regions of all four pairs of acrocentric autosomes (Fig. 5a, b). On polytene chromosomes, CDSTR138 signals were detected at the chromocenter (Fig. 5f). The CDSTR230 mapped exclusively to a single non-centromeric locus on one arm of the metacentric Y chromosome (Fig. 5d). Finally, the CDSTR177 mapped to the pericentromeric region of all acrocentric autosomes, to the microchromosomes, and also at the most distal end of the X heterochromatic block (Fig. 5c). In polytene chromosomes, a single CDSTR177 signal was detected in the euchromatin of one chromosome arm and on the chromocenter (Fig. 5h). We did not perform FISH for DBC-150 because its chromosomal location was previously characterized, revealing and exclusive localization to the microchromosomes (Kuhn et al. 2007).

The CDSTR8, CDSTR138 and CDSTR177 satDNAs were all mapped to the pericentromeric regions of the four pairs of acrocentric chromosomes, and to the microchromosomes. In the four pairs of acrocentric chromosomes, double FISH experiments using CDSTR8/CDSTR138 (Fig. 5a,b) and CDSTR8/CDSTR177 (Fig. 5c) show that these satDNA co-occur in the pericentromeric heterochromatin, with partially overlapping signals. Notably, CDSTR138 appears to be more proximally located relative to the centromere than either CDSTR8 or CDSTR177.

### Transcriptional analysis of satellite DNAs

**Fig. 6:**
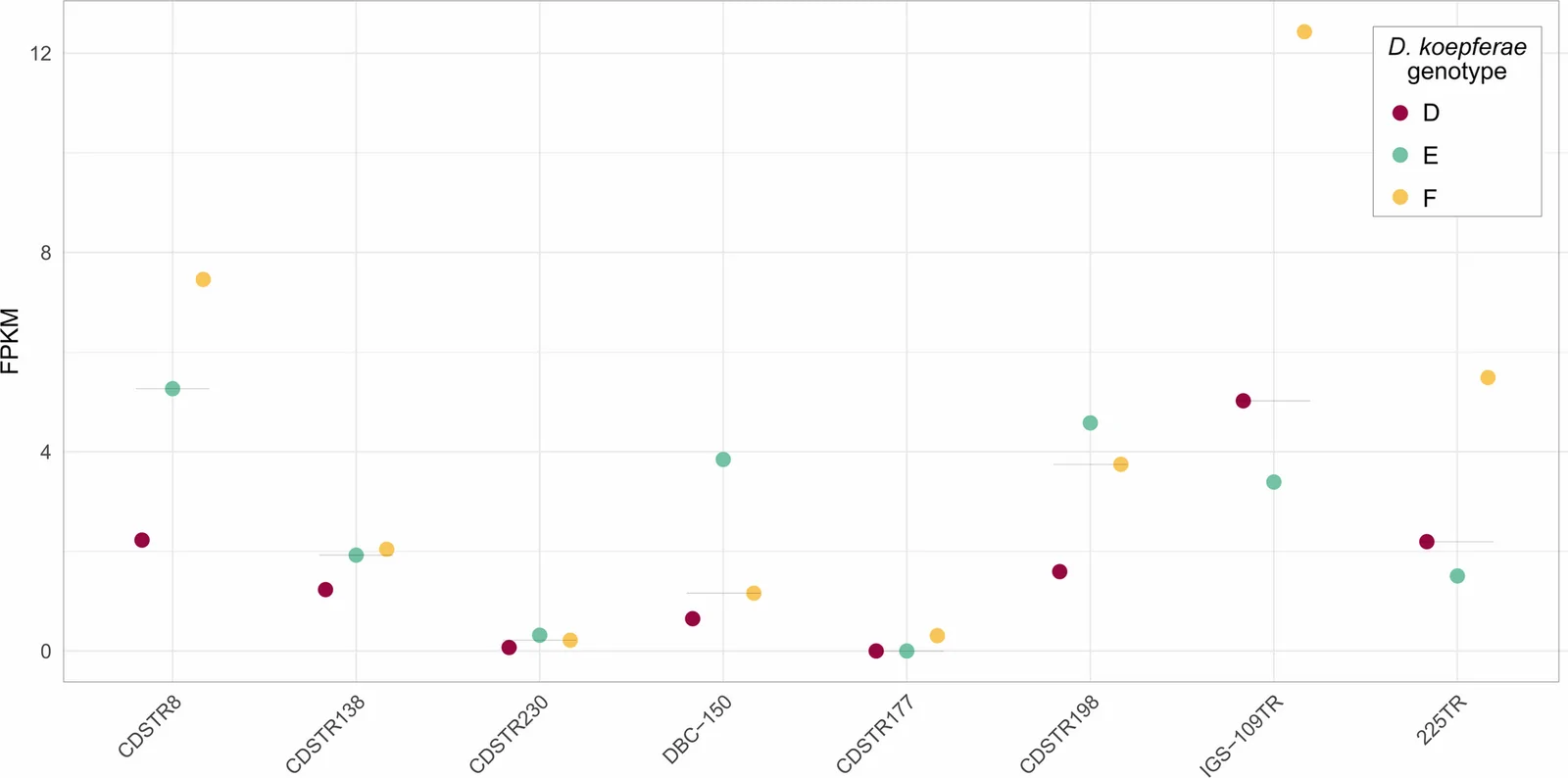
Transcription profile of the satellite DNAs (CDSTR8, CDSTR138, CDSTR230, DBC-150, CDSTR177), the minisatellite DNA (CDSTR198) and rDNA (IGS-109TR and 225TR) of *D. koepferae*. The three genotypes represent biological replicates (De Panis et al. 2022). Counts were normalized to one million reads

Using publicly available *D. koepferae* RNA-seq data, (De Panis et al. 2022) we detected transcripts derived from all five satDNA families identified in this species (CDSTR8, CDSTR138, CDSTR230, DBC-150 and CDSTR177) (Fig. 6). Expression levels were generally low (FPKM values ranging from 0 to 7.46), with CDSTR8 showing the highest values (2.23–7.46 FPKM), followed by CDSTR138 (1.23–2.04 FPKM) and DBC-150 (0.65–3.85 FPKM), whereas CDSTR230 (0.07–0.32 FPKM) and CDSTR177 (0–0.31 FPKM) were consistently detected at minimal levels. Although all satDNAs were transcriptionally active in the three genotypes analyzed, quantitative differences were observed among them, particularly for CDSTR8. As the three genotypes are known to differ in chromosomal organization (De Panis et al. 2022), such variation may reflect underlying genomic or chromatin differences. However, the available data does not allow further inference. For comparison, the euchromatic minisatellite CDSTR198 exhibited higher transcript levels than the satDNAs (FPKM ranged from 1.59 to 4.58), consistent with a more permissive chromatin environment (Fig. 6). The ribosomal DNA spacers IGS-109TR and 225TR displayed substantially higher transcript levels (FPKM ranged from 0.51 to 5.49 and from 3.39 to 12.43, respectively) than both satDNAs and the minisatellite DNA, particularly IGS-109TR, which reached the highest values observed in the dataset (Fig. 6). This pattern is consistent with their association with the highly active rDNA locus.

## Discussion

The *buzzatii* cluster, within the large *repleta* group, has been extensively investigated concerning satDNA organization and evolution (reviewed by Kuhn et al. 2021). These studies first started with the identification of satDNA families through genomic DNA digestion with restriction enzymes and more recently through the analysis of sequenced genomes. While the satDNA composition of six species from the *buzzatii* cluster has been already well characterized, *D. koepferae* is the only species from the cluster where satDNAs have not been investigated from sequenced genomes. Here we present the first identification and characterization of satDNAs of *D. koepferae,* based on the analysis of sequenced genomes of two populations, one from Argentina (DkoeA) and one from Bolivia (DkoeB).

From the five satDNAs identified in *D. koepferae* (CDSTR8, CDSTR138, CDSTR230, DBC-150 and CDSTR177), four satDNAs are shared with other species of the *buzzatii* cluster, while CDSTR177 is described here for the first time and is species-specific to *D. koepferae.* In particular, the CDSTR8 repeat length is shorter than the > 100 bp typically reported for satDNAs in the *repleta* group (Kuhn et al. 2021), even though it is organized in the form of higher-order repeats, that can reach up to 48 bp (i.e. 6-mer repeats), partially converging toward this range.

The total satDNA contribution in *D. koepferae* was lower (0.8–0.9%) compared to what is typically found in *Drosophila*, where satDNA often contributed with > 10% of the genomic DNA (Wei et al. 2014). On the other hand, such low satDNA genomic contribution (< 5%) has also been consistently reported in other *Drosophila* species from the *repleta* group, what is probably related to the presumably low heterochromatin content present in species within this group (Bosco et al. 2007; Kuhn et al. 2021; Laborne et al. 2024; Rossi et al. 2025). The satDNA content of *D. koepferae* is also substantially lower than that reported for its closest relatives within the *buzzatii* cluster (~ 4%; Lima et al. 2017; Laborne et al. 2024; Rossi et al. 2025). As all these estimates were generated using the same methodology and passed through equivalent quality-control filters, this difference in satDNA content likely reflects a biological difference rather than a technical artifact.

**Fig. 7:**
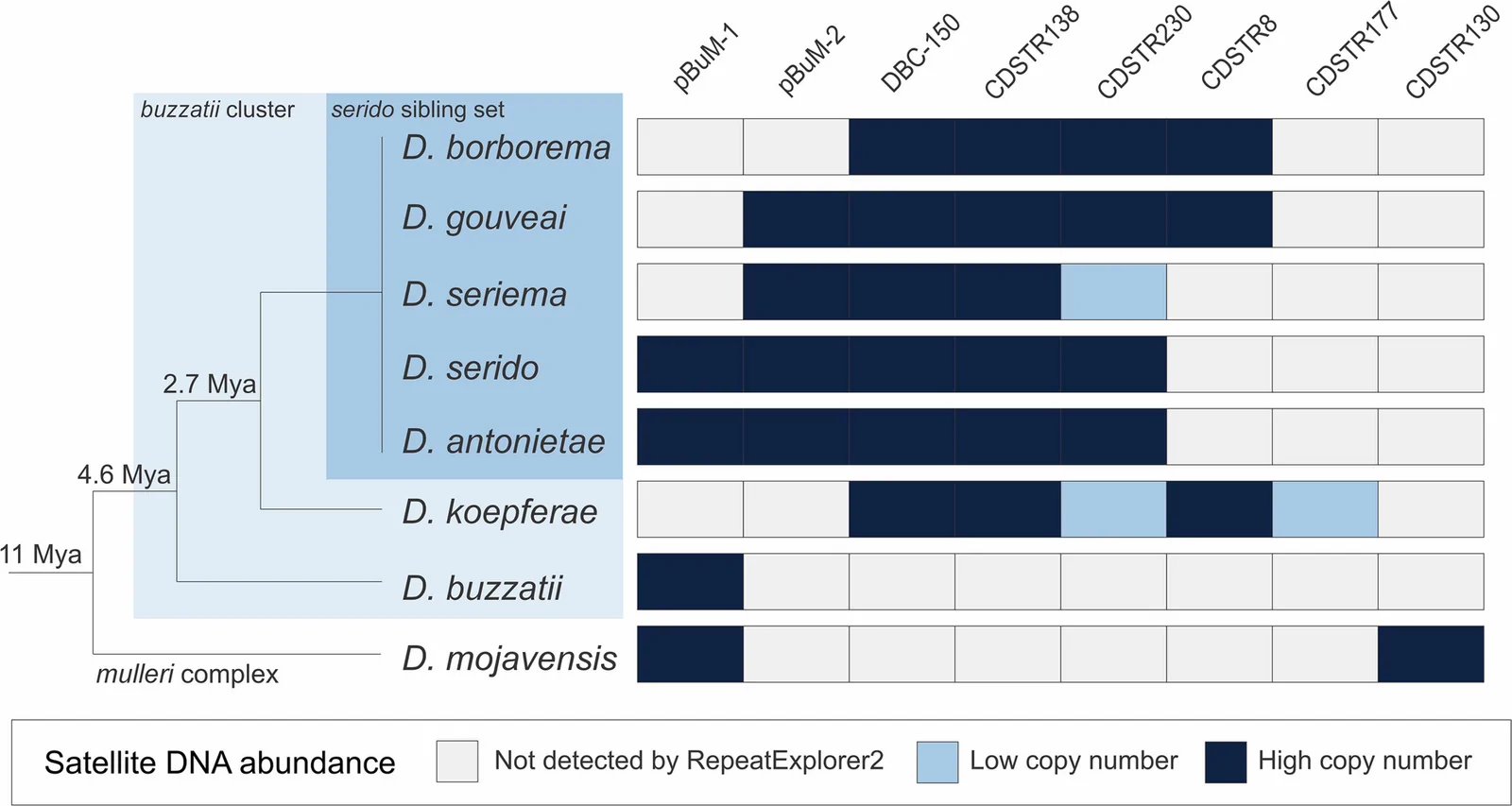
Overall profile of satellite DNAs identified in species of the *buzzatii* cluster, with *D. mojavensis* shown as an outgroup

Interestingly, the pBuM satDNA (pBuM-1 and pBuM-2 subfamilies) was not detected in any repetitive cluster of *D. koepferae* returned by RepeatExplorer2/TAREAN. The pBuM satDNA has been previously reported in high copy numbers (pBuM-1, pBuM-2, or both) in all species from the *buzzatii* cluster, except *D. borborema* (Fig. 7; Rossi et al. 2025), and even in *D. mojavensis*, which belongs to the *mojavensis* complex, also within the *repleta* group. Direct BLASTn searches against the genome assemblies, however, showed that pBuM is present in both *D. koepferae* populations, but in a small number of isolated copies, with no evidence of tandem organization. These results confirm previous findings based on DNA-DNA hybridization (Kuhn and Sene 2005) and FISH in mitotic chromosomes and DNA fibers (Kuhn et al. 2008).

Satellite DNAs were classically proposed to originate through tandem amplification of an ancestral sequence driven by unequal crossing-over (Smith 1976). Although virtually any sequence, particularly non-coding DNA, may undergo tandem expansion, increasing evidence indicates that several satDNAs have originated from full-length or partial sequences of transposable elements (Meštrović et al. 2015; Paço et al. 2019; Zattera and Bruschi 2022; Šatović-Vukšić et al. 2025). In *Drosophila*, there are several examples of satDNAs that originated from the amplification of internal tandem repeats that are integral parts of transposable elements. In *D. virilis* for example, the origin of two satDNAs, TIR-220 and 154TR, have been traced back to the amplification of internal tandem repeats present in two different transposable elements from the “cut-and-paste” and “Helentrons” superfamilies of Class II (Dias et al. 2014, 2015). Amplification of internal tandem repeats of Helentrons has also been recently reported in *Drosophila* species from the *montium* group (Silva et al. 2023).

Here, we found that CDSTR177 share homology to the *Galileo* transposable element. *Galileo* is a well characterized Foldback-like transposon from the *P* superfamily, known for its long terminal inverted repeats (TIRs) and inherent genomic instability, properties which facilitate recombination and the generation of different types of chromosomal rearrangements, including inversions (Lobachev et al. 1998; Zhou et al. 2001; Casals et al. 2003). Interestingly, the homology between CDSTR177 and *Galileo* lies in the central “M” region, where no tandem repeats exist. In this context, two scenarios could explain this association: (i) CDSTR177 originated from in situ tandem amplification of a unique sequence within the *Galileo* element, similar to the origin of TIR-220 and 154TR from internal tandem repeats of Class II transposons in *D. virilis* (Dias et al. 2014, 2015); or (ii) a segment of a pre-existing satellite DNA was captured by *Galileo* and subsequently dispersed to multiple, independent genomic loci through transposition, as reported for the TCAST satellite in *Tribolium castaneum*, where retrotransposon-mediated capture and mobilization of TCAST-derived sequences produced dispersed loci with variable copy number, from partial monomers to tandem arrays (Brajković et al. 2012).

To distinguish between these scenarios, we examined the genomic context of CDSTR177 and *Galileo* copies identified in the assembled genomes of both populations. In particular, loci containing a single CDSTR177 monomer and loci containing multiple monomers in tandem array (Fig. 4b, c) shared identical flanking *Galileo* sequence, indicating that the array most likely arose through progressive in situ amplification of a single ancestral CDSTR177-like segment within a specific *Galileo* copy, rather than through independent capture and dispersal of a pre-existing satellite sequence or array across multiple, unrelated *Galileo* copies. As *Galileo* is present in other species from the *buzzatii* cluster but CDSTR177 is not, we conclude that the amplification event leading to this satDNA occurred after the divergence of the *D. koepferae* lineage from the remaining species of the *buzzatii* cluster.

In *D. koepferae*, the CDSTR138 satDNA appears to be more proximally located relative to the centromere than the other satDNAs. In all other species from the *buzzatii* cluster, except *D. buzzatii* that lacks this satDNA, CDSTR138 has also been consistently found closer to the centromeres than the other satDNAs (Laborne et al. 2024). Accordingly, we found that CDSTR138 in *D. koepferae* also presents features commonly associated to centromeric satDNAs in eukaryotes (Prytkova et al. 2011; Struhl and Segal 2013; Talbert and Henikoff 2020; Kuhn et al. 2021), such as the ability to form stable secondary structures and display AA/TT/AT nucleotide periodicity (Laborne et al. 2024; Fig. S10).

No satDNA was mapped to the (peri)centromeric region of the X or Y chromosomes, suggesting that these chromosomes either lack large satDNA arrays or contain low-copy, chromosome-specific repeats, that did not meet our classification criteria. In the sex chromosomes of other species from the *buzzatii* cluster, satDNAs have only be found in the pericentromeric regions of the Y chromosome of *D. buzzatii* and of the X chromosomes of *D. antonietae*, *D. serido*, *D. gouveai* and *D. borborema* (Kuhn et al. 2008; Lima et al. 2017; Laborne et al. 2024; Rossi et al. 2025). Other repetitive DNA associated to the centromeric DNA of the sex chromosomes in these species are a group of non-autonomous Helentrons elements called “PERI” (Kuhn and Heslop-Harrison 2011) and ribosomal genes (Laborne et al. 2024).

Once dismissed as transcriptionally silent due to its repetitive, heterochromatic nature, satDNAs are now known to be actively transcribed across eukaryotes, from yeast to mammals (Volpe et al. 2002; Topp et al. 2004; Pezer and Ugarković 2008; Grewal and Elgin 2007; Rudert et al. 1995; Chan et al. 2012; McNulty et al. 2017). In *Drosophila*, studies demonstrate that satellite transcripts are required for proper kinetochore and heterochromatin formation, recruitment of CENP-A and faithful chromosome segregation (Usakin et al. 2007; Rošić et al. 2014; Bobkov et al. 2018; Ghosh and Lehner 2022).

Here, we detected RNA-seq reads mapping to the consensus sequences of all five satDNAs identified in *D. koepferae*; however, their expression was low relative to repetitive elements embedded within the rDNA locus, and lower than that of the euchromatic CDSTR198 minisatellite. This elevated transcription of CDSTR198 is consistent with a more permissive chromatin environment typical of euchromatin. These transcriptomic data were obtained from total RNA libraries (De Panis et al. 2022) and are therefore not restricted to polyadenylated transcripts. The number of mapped reads are consistent with low-level satDNA transcription, but this inference should be treated with caution since we cannot rule out that a small fraction of mapped reads originates from residual genomic DNA. Moreover, the libraries were generated from a single larval stage (De Panis et al. 2022), therefore we cannot exclude the possibility that these satDNAs are expressed differently in other developmental stages. In this context, direct chromatin profiling approaches such as ATAC-seq to assess genome-wide accessibility (Shatskikh et al. 2020), MNase-seq to map nucleosome positioning (Chereji et al. 2019) or histone ChIP-seq to characterize repressive and active marks (Henikoff et al. 2014; Shatskikh et al. 2020; Wei et al. 2021) would provide valuable evidence to test the chromatin state differences inferred here between CDSTR198 and the heterochromatic satDNAs.

We also found that CDSTR8 is expressed at relatively higher levels than CDSTR138. In mouse, for example, centromeric satDNA transcripts are typically maintained at low levels and undergo rapid turnover, primarily functioning to ensure kinetochore formation and proper cell division (Smurova and Wulf 2018; Talbert and Henikoff 2018). Also, pericentromeric satDNAs are often transcribed at higher levels, since they are critical for the formation and maintenance of the heterochromatin architecture (Smurova and Wulf 2018; Baumann et al. 2023). Whether such functional differences exist in *Drosophila* is not yet clear, nevertheless, it is plausible that the relatively higher expression of CDSTR8 relative to CDSTR138 may be related to their functional roles at the pericentromeric and centromeric regions, respectively.

Previous studies revealed incipient differentiation between Argentinean and Bolivian allopatric populations of *D. koepferae*, based on chromosomal inversions and allozyme markers (Fontdevila et al. 1988), mitochondrial genomes (Moreyra et al. 2019) and nuclear genomic data (Moreyra et al. 2023). Despite these differences, the two populations remain fully cross-compatible in lab conditions (Fontdevila et al. 1988). Divergence time estimates suggest that population separation occurred approximately between 0.3 to 0.7Mya (Moreyra et al. 2023). Here, we detected statistically significant differences in the relative abundance of the three CDSTR8 variants (A, B and C) between the two populations (Fig. 2b). ML trees based on DBC-150, CDSTR138, CDSTR177 and CDSTR230 revealed clear inter-population differentiation only for CDSTR230 (Fig. 3). Notably, the CDSTR230 was mapped exclusively to the Y chromosome of *D. koepferae*. Unlike autosomes and the X chromosome, the Y chromosome is largely non-recombining and has a reduced effective population size relative to autosomes and the X chromosome. These features enhance the impact of genetic drift and accelerate lineage sorting, making Y-linked sequences particularly prone to rapid population differentiation. In summary, CDSTR8 and CDSTR230 satDNAs provide additional evidence for inter-population differentiation between Argentinean and Bolivian populations of *D. koepferae*.

The *buzzatii* cluster comprises seven closely related cactus-breeding species. All but one (*D. buzzatii*) are endemic to South America (Manfrin and Sene 2006). Based on early works on morphological aspects of the male genitalia, the cluster was divided into two groups: one composed of *D. buzzatii* and the other composed by the remaining species that were grouped into the ‘*D*. *serido* sibling set’ (reviewed by Manfrin and Sene 2006). While this division has been largely corroborated by several genetic markers, the phylogenetic position of *D. koepferae* within the *buzzatii* cluster is still debatable: mitochondrial DNA markers suggest a sister relationship with *D. buzzatii* (Manfrin et al. 2001; Franco and Manfrin 2012; Moreyra et al. 2019), while nuclear DNA markers place *D. koepferae* either within or as a sister species to the ‘*D*. *serido* sibling set’ (Rodriguez-Trelles et al. 2000; Hurtado et al. 2019; Moreyra et al 2023).

The rapid divergence and species-specific fixation of satDNA families make them valuable markers for taxonomy and phylogenetics, particularly among closely related or cryptic taxa (Biscotti et al. 2015; Garrido-Ramos 2017; Kuhn et al. 2021; Despot-Slade et al. 2022). For example, in European brown frogs the S1 satDNA shows interspecific differences in monomer length, sequence, copy number and chromosomal distribution, allowing it to serve as an useful marker to distinguish morphologically cryptic species (Picariello et al. 2002); in oysters, a conserved centromeric satellite supported discrimination between *Ostrea edulis* and *O. stentina* (López-Flores et al. 2004) and in American marsupialis, satDNAs recovered species-level phylogenetic relationships congruent with previous studies (Dias et al. 2021).

Building on this framework, the comprehensive satDNA profile of *D. koepferae* provides useful data for the discussion on the phylogenetic position of the species within the *buzzatii* cluster (Fig. 7). *D. koepferae* shares four satDNAs with species from the ‘*D*. *serido* sibling set’. Furthermore, the most abundant satDNA in *D. buzzatii*, the pBuM-1, was not detected in any repetitive cluster of *D. koepferae*. These satDNA patterns are consistent with a closer phylogenetic position of *D. koepferae* relative to the ‘*D. serido* sibling set’, as suggested by nuclear markers (Rodriguez-Trelles et al. 2000; Hurtado et al. 2019; Moreyra et al. 2023) and earlier morphological and biogeographic data (Tidon-Sklorz and Sene 1995). Accordingly, the closer phylogenetic relationship between *D. buzzatii* and *D. koepferae* revealed by mtDNA is likely the result of hybridization between the species in the past (Manfrin and Sene 2006; Moreyra et al. 2019). In fact, both species are found in sympatry in Argentina and Bolivia (Manfrin and Sene 2006).

Notably, *D. borborema* is the species that shares more satDNAs (DBC-150, CDSTR138, CDSTR230 and CDSTR8) with *D. koepferae*. Nevertheless, a close phylogenetic association between these species has not been reported by any other genetic marker. In order to get more insights into the phylogenetic relationship between *D. koepferae* and the ‘*D*. *serido* sibling set’, we constructed an ML tree based on the Y-linked CDSTR230 satDNA, that is present in all species from the *buzzatii* cluster and shows signatures of population (this work) and species differentiation (Laborne et al. 2024). The resulting ML clearly shows “concerted evolution”, as repeats from the same species form species-specific branches, separated from repeats from other species (Fig. S11). Furthermore, the ML tree shows *D. koepferae* CDSTR230 repeats clearly clustered separately from the CDSTR230 repeats from the other species of the *buzzatii* cluster. In summary, the CDSTR230 tree place *D. koepferae* in a basal position within the ´*D. serido* sibling set’. This basal placement inferred from the satDNA tree aligns with hypotheses proposed by Tidon-Sklorz and Sene (1995), based on differences in aedeagi morphological types and metaphasic chromosome patterns. Moreover, the geographic distribution reflects this divergence: *D. koepferae* inhabits the Argentinean Chaco, whereas the other species from ‘*D. serido* sibling set’ are present in different Brazilian biomes (e.g. Caatinga, Cerrado and Rocky Fields) (Tidon-Sklorz and Sene 1995).

Assuming that the phylogeny depicted in Fig. 7 reflects the true evolutionary relationship among species (even though the internal relationships within ´*D. serido* sibling set’ are not solved and depicted as a polytomy), it also reveals a rapid evolutionary turnover of satDNAs within the last 2My, including independent losses of pBuM in *D. koepferae* and *D. borborema* and independent maintenance of CDSTR8 in *D. koepferae* and in the lineage leading to *D. borborema* and *D. gouveai*. Furthermore, a new emerging satDNA (CDSTR177) was only detected in *D. koepferae*. The turnover of satDNAs is thought to arise primarily from neutral, recombination or replication-mediated processes, such as unequal crossing-over and replication slippage, which can promote both the expansion and contraction of satDNA tandem arrays (Sproul et al. 2020a, b; Lauria Sneideman and Meller 2021; Thakur et al. 2021). Rolling circle amplification of extrachromosomal circular DNA containing satDNA repeats excised by recombination within arrays, followed by and reinsertion into the chromosomes (Feliciello et al. 2005; Sproul et al. 2020a, b; Lauria Sneideman and Meller 2021), and reverse transcription of satDNA transcripts followed by reintegration into genome are additional processes that can result satDNA expansions (Bersani et al. 2015). Concerning the centromeres, pBuM is the centromeric satDNA in *D. buzzatii*, while in the other species, pBuM (when present) is located more distally to the centromeres. In the ´*D. serido* sibling set’, CDSTR138 is the main centromeric component, while in *D. buzzatii*, this satDNA has not been found. These findings emphasize the fundamental role of rapid satDNA turnover in shaping heterochromatin and centromeric architecture in recently radiated species groups (Courret et al. 2024; Gebert et al. 2025).

In conclusion, our study delivers the first comprehensive characterization of the satDNA landscape of *D. koepferae*, establishing a robust framework for investigating key aspects of satDNA biology and evolution, and for advancing our understanding of the evolutionary history and phylogenetic relationships of *D. koepferae* within the *buzzatii* cluster.

## Supplementary Information

Below is the link to the electronic supplementary material.

Supplementary file1 (PDF 2.24 MB)

Supplementary file2 (PDF 929 KB)

Supplementary file3 (PDF 133 KB)

## Data Availability

For genomic analyses, we used publicly available (NCBI) raw Illumina reads from sequenced genomes of *Drosophila koepferae* (SRX12630073 and SRX18356637), and raw PacBio reads (SRR22206171 and SRR22206170) and the corresponding assembled genomes (GCA_030914325.1 and GCA_030914345.1). For transcriptional analysis, we used publicly available (NCBI) raw RNAseq reads from *D. koepferae * PRJNA858943.
