# Supplementary material for: Satellite DNAs in Drosophila koepferae (repleta group) reveal patterns of origin, chromosomal organization, transcription, and turnover in the buzzatii cluster
Source: Chromosome Res. 2026 Sep 8;34(1):22. doi: 10.1007/s10577-026-09814-5 (PMC13553610; doi:10.1007/s10577-026-09814-5)
Supplement: Supplementary file 1 [file 10577_2026_9814_MOESM1_ESM.pdf]

Satellite DNAs in *Drosophila koepferae* (*repleta* group) reveal patterns of origin, chromosomal organization, transcription, and turnover in the *buzzatii* cluster.

Ana Mattioli Laborne, John S. Heslop-Harrison and Gustavo C.S. Kuhn

Supplementary Material – Figures

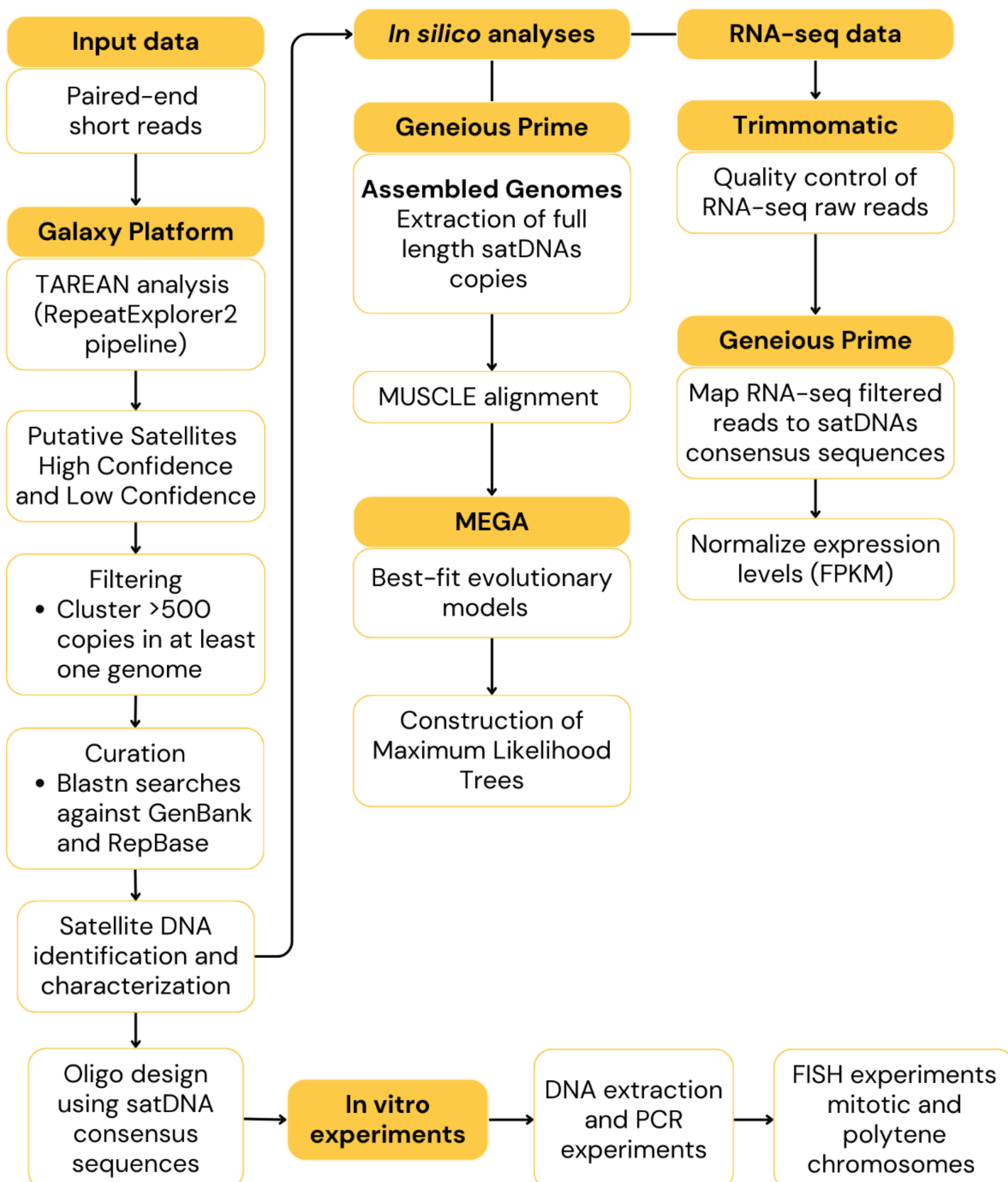

Fig. S1 Workflow used for satellite DNA (satDNA) identification and analyses.

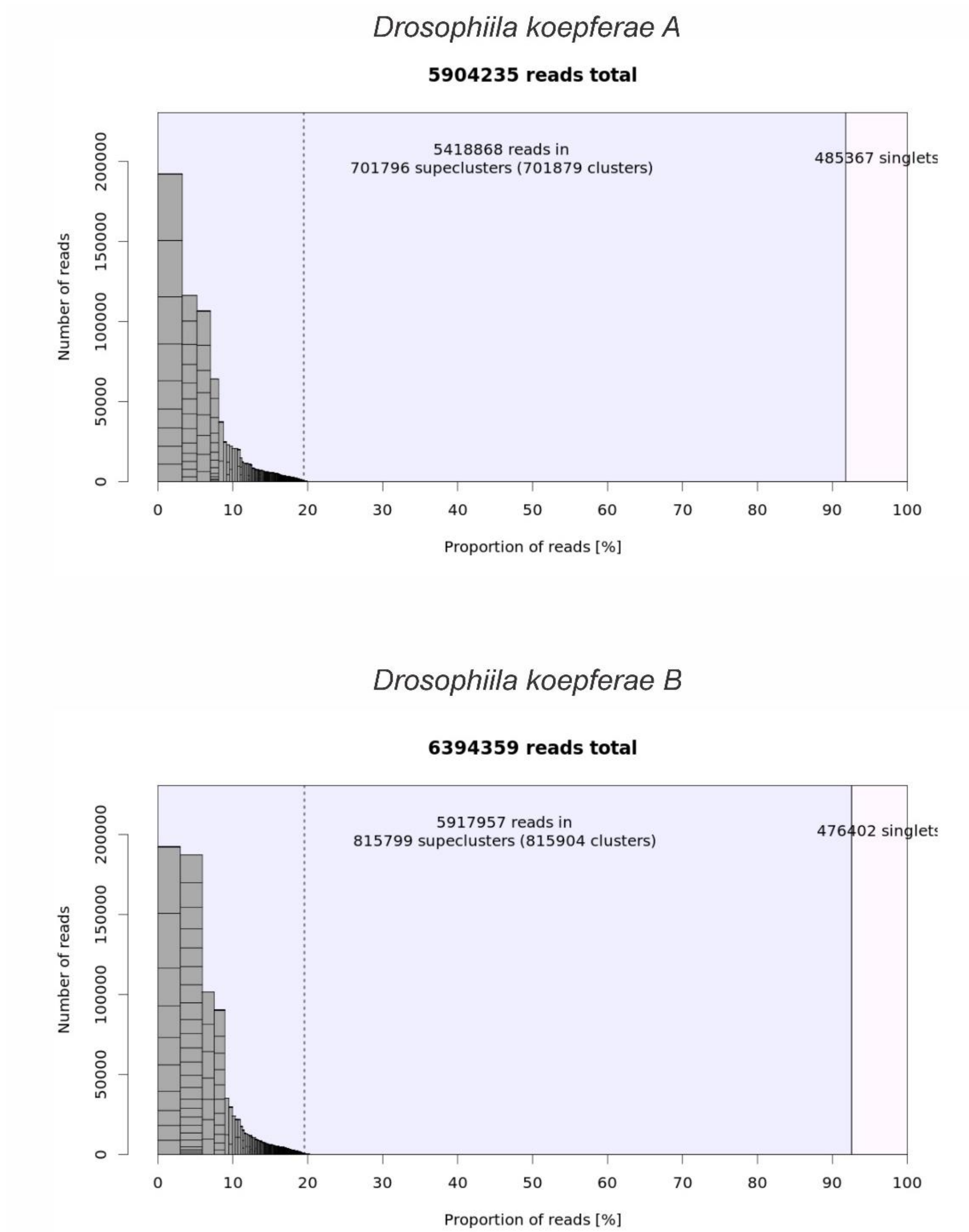

Fig. S2 Graphical summary of RepeatExplorer2/TAREAN analysis in the two *Drosophila koepferae* populations (Argentinean: up; and Bolivian: down). Bars represent superclusters, height indicates read numbers and width their proportion among all analyzed reads. Each cluster is represented by the rectangles within the bars.

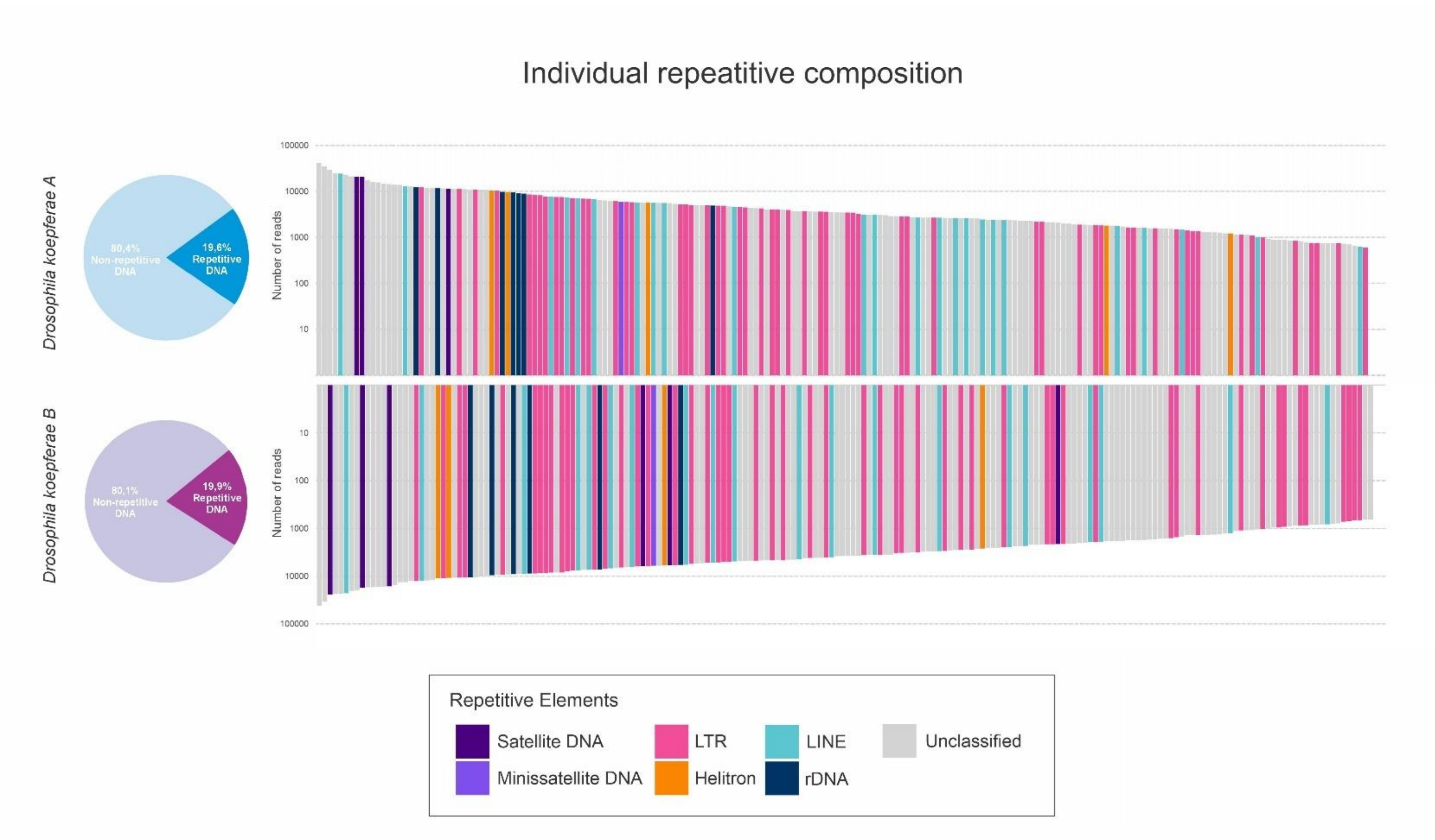

Fig. S3 The individual repetitive DNA landscape of the two populations of *D. koepferae* (A and B).

CDSTR138

Drosophila koepferae A

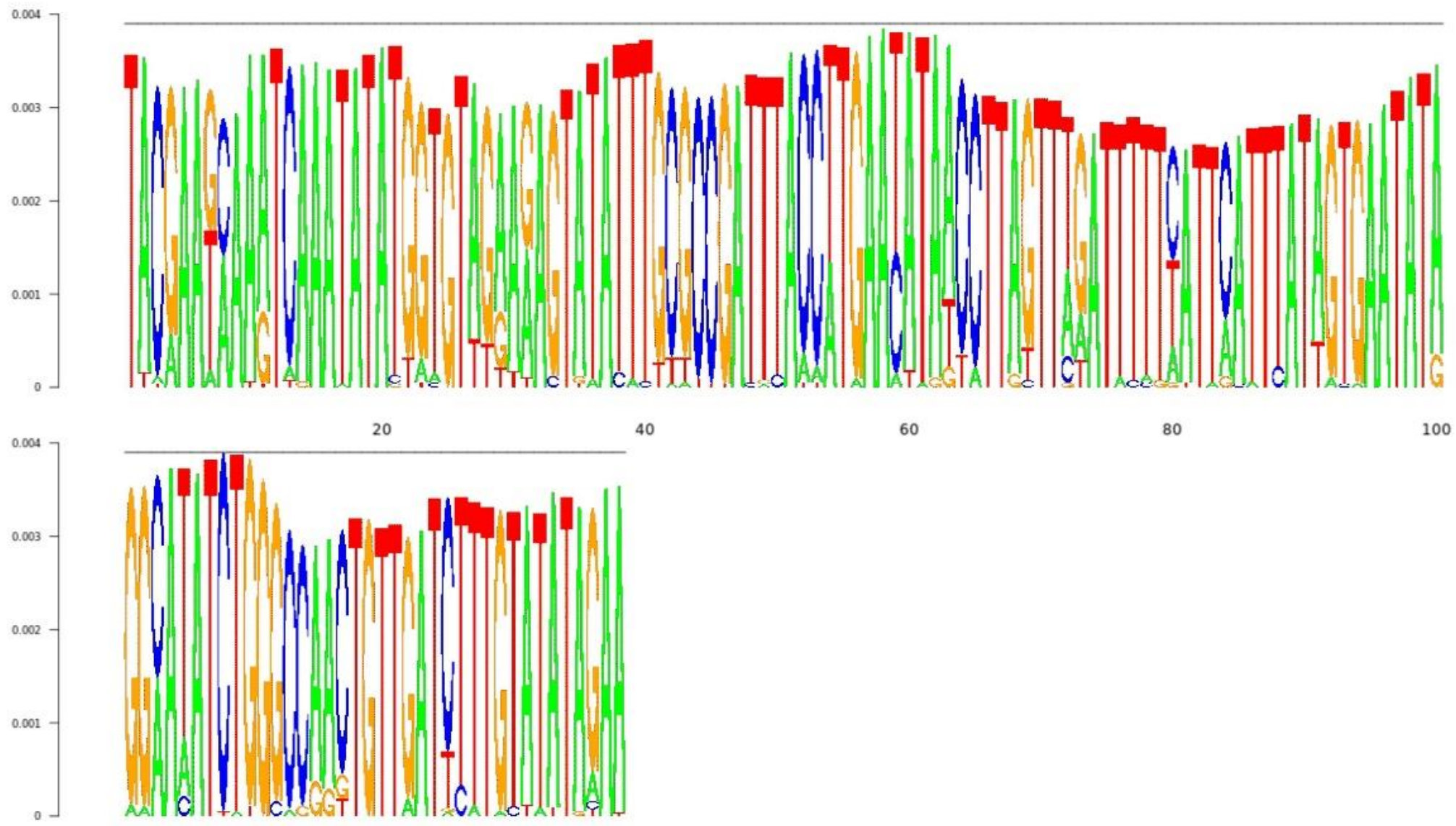

Drosophila koepferae B

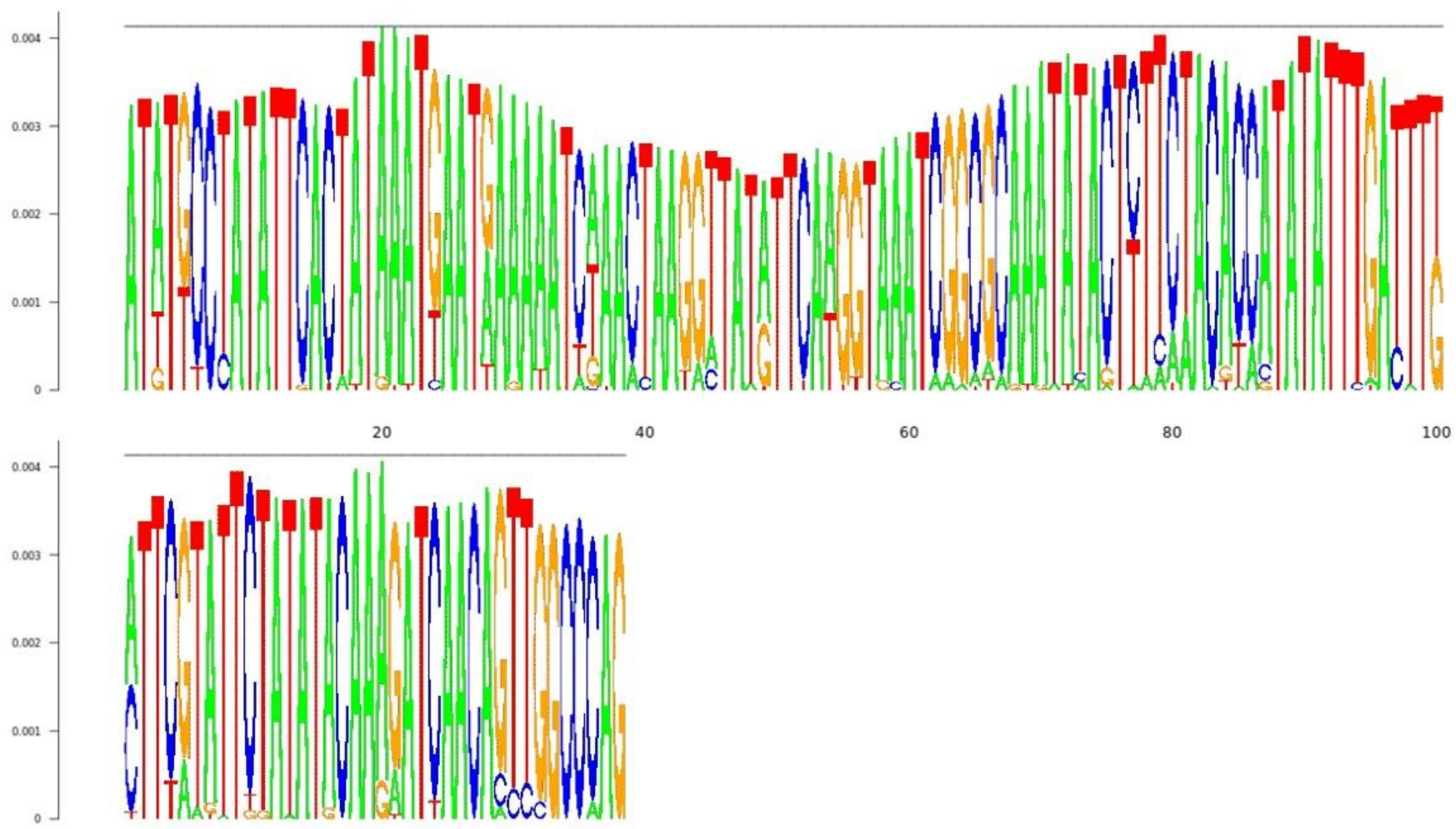

Fig. S4 Sequence logo generated by TAREAN pipeline for CDSTR138 in both *D. koepferae* populations (Argentinean and Bolivian).

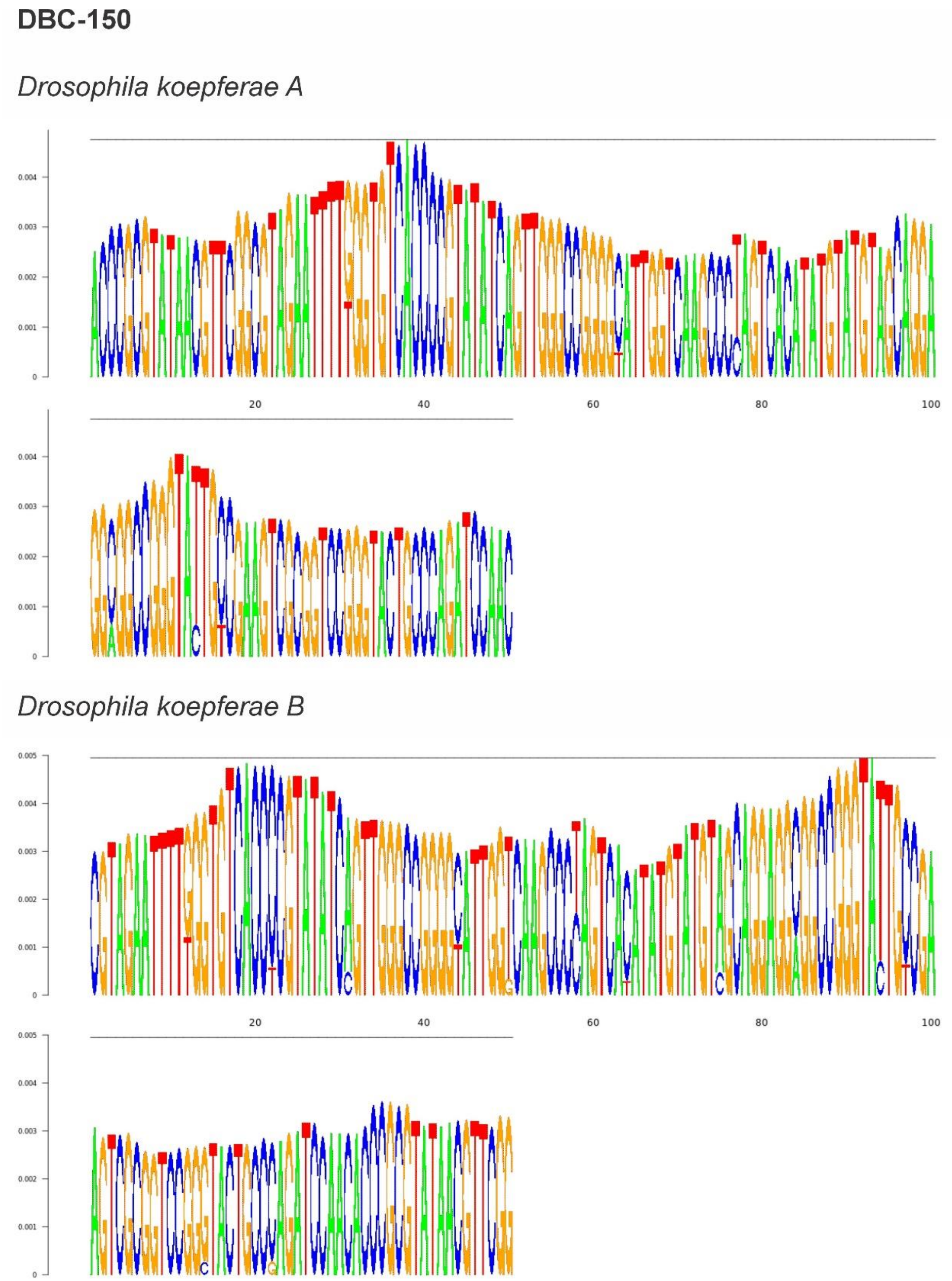

Fig. S5 Sequence logo generated by TAREAN pipeline for DBC-150 in both *D. koepferae* populations (Argentinean and Bolivian).

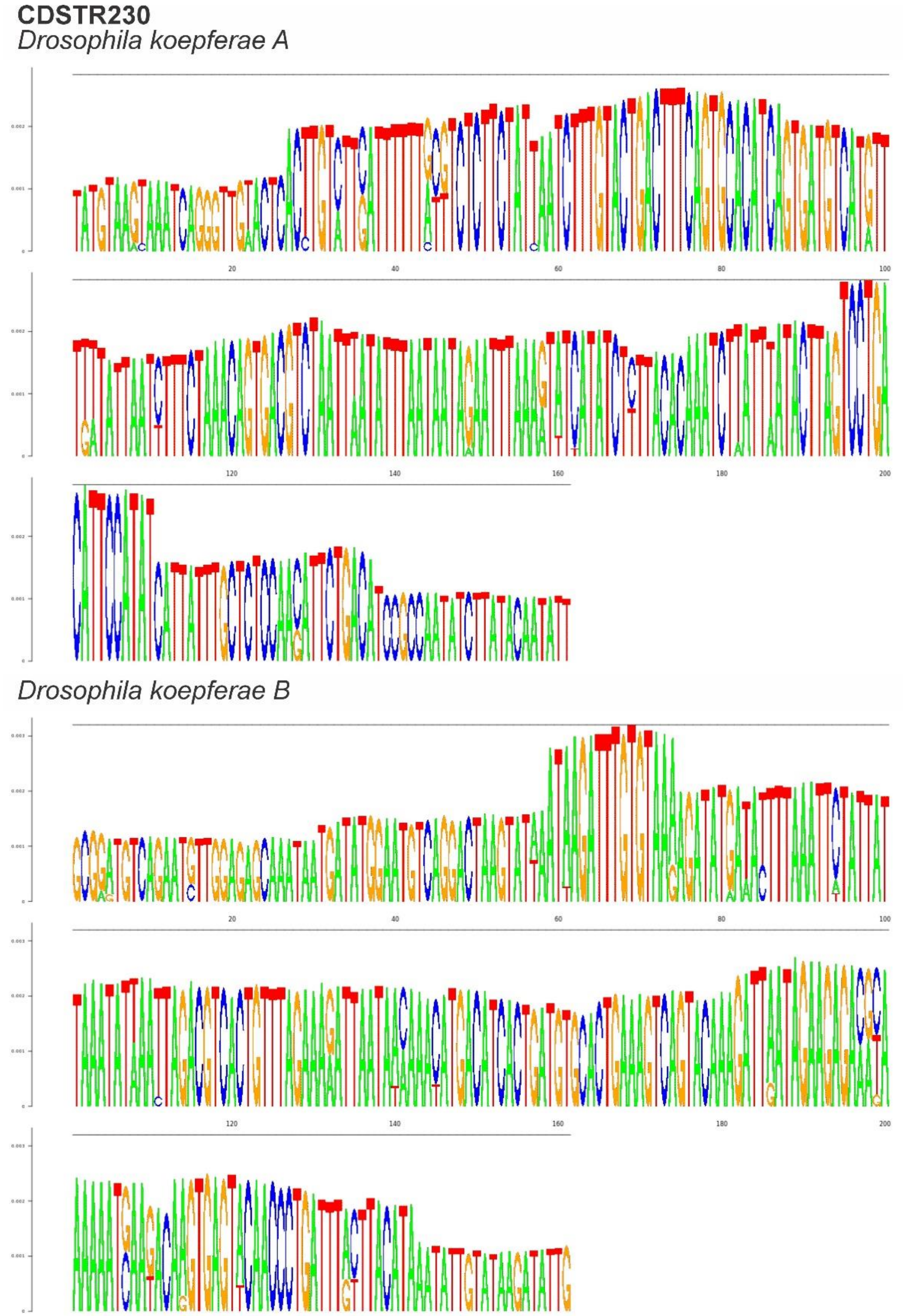

Fig. S6 Sequence logo generated by TAREAN pipeline for CDSTR230 in both *D. koepferae* populations (Argentinean and Bolivian).

CDSTR177

Drosophila koepferae A

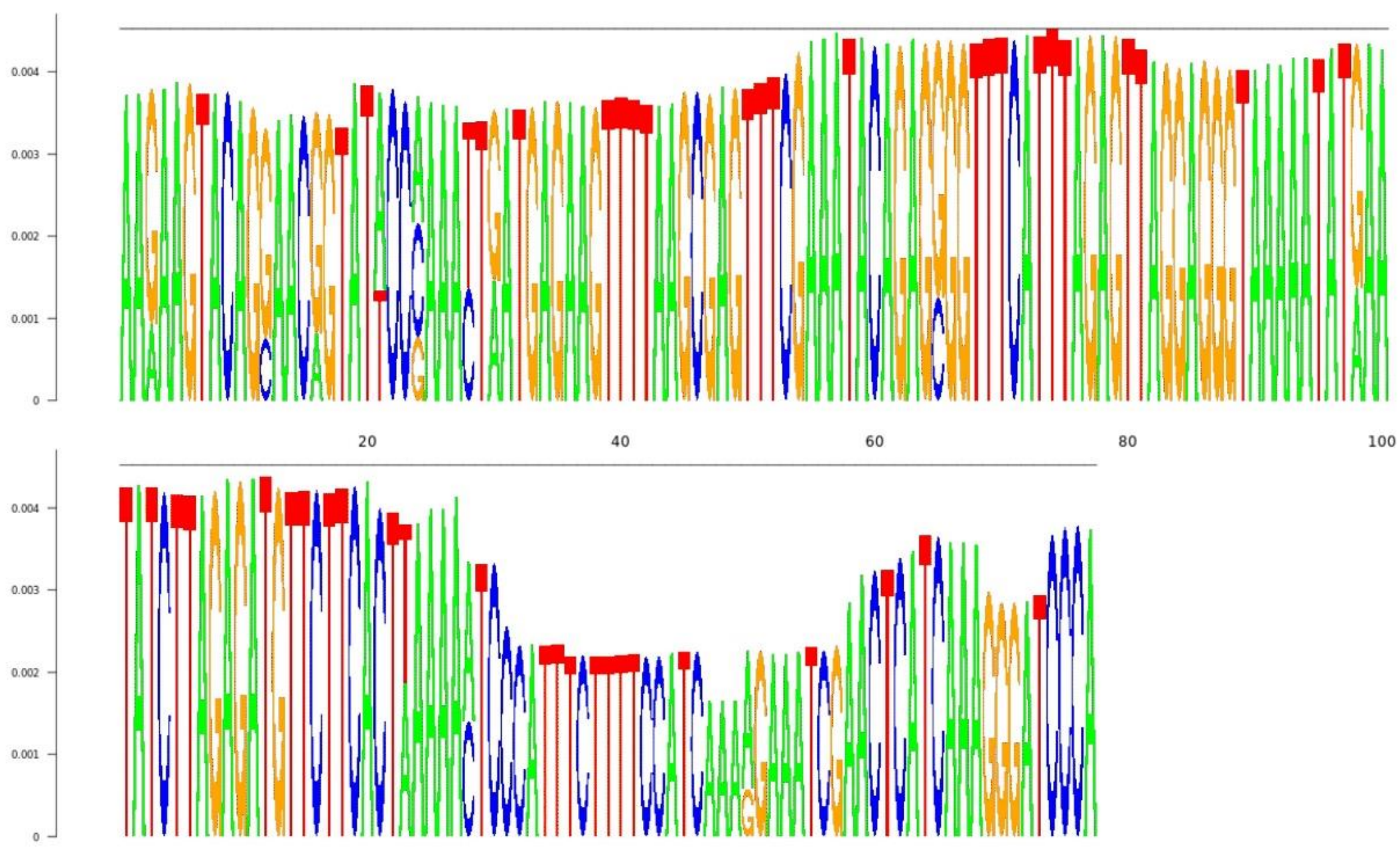

Drosophila koepferae B

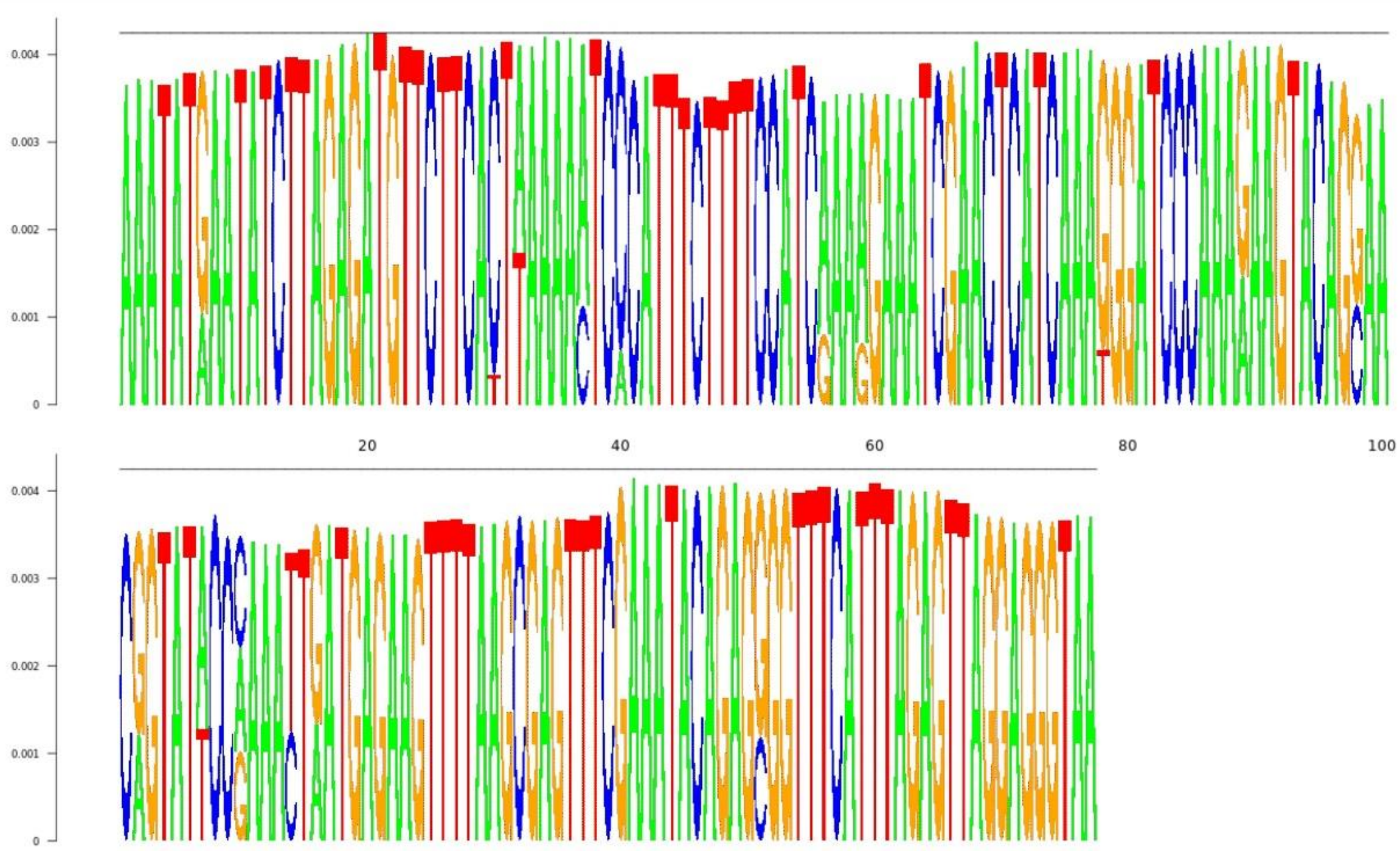

Fig. S7 Sequence logo generated by TAREAN pipeline for CDSTR177 in both *D. koepferae* populations (Argentinean and Bolivian).

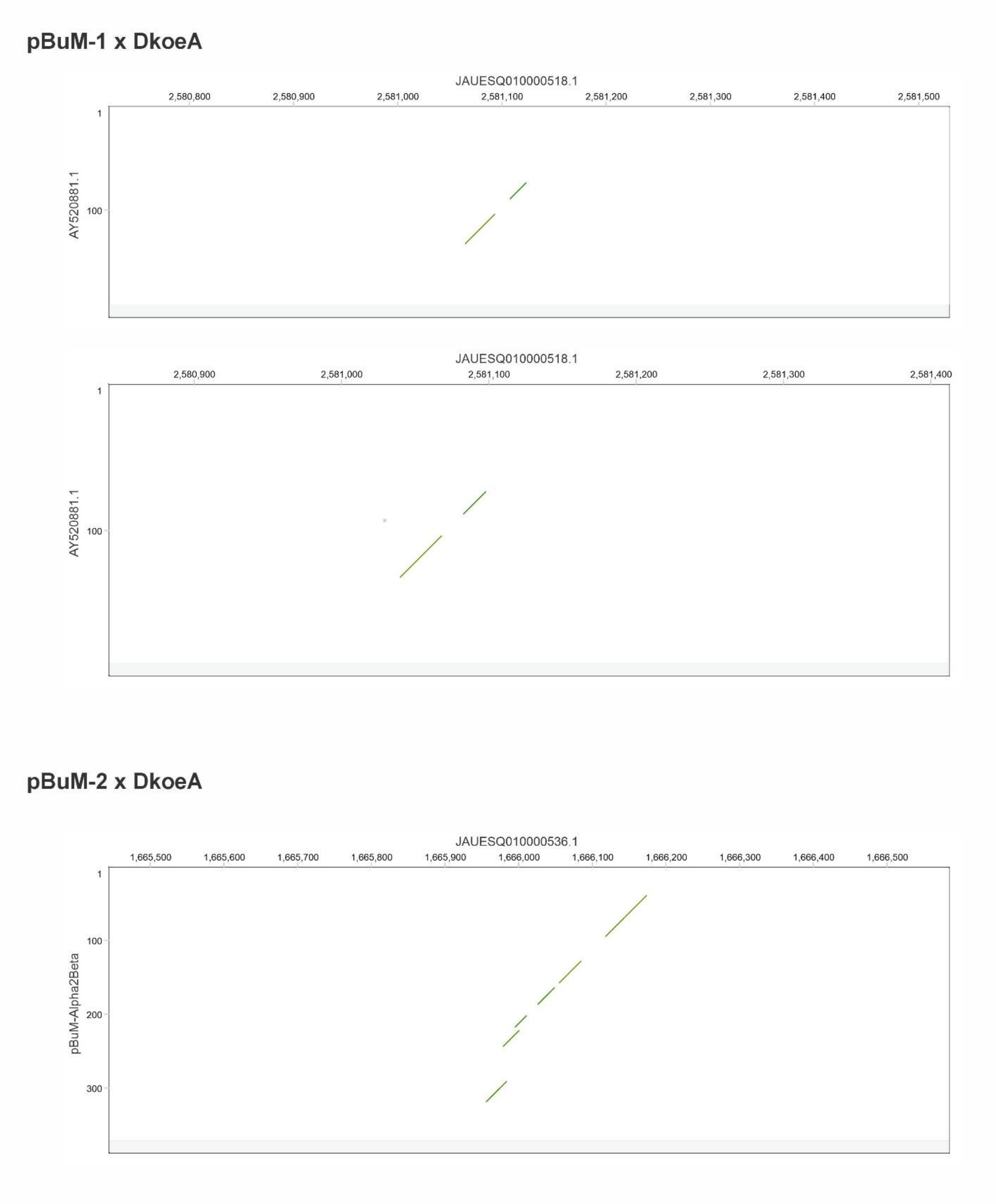

Fig. S8 Dot-plots showing the presence of pBuM in *Drosophila koepferae* Argentinean population.

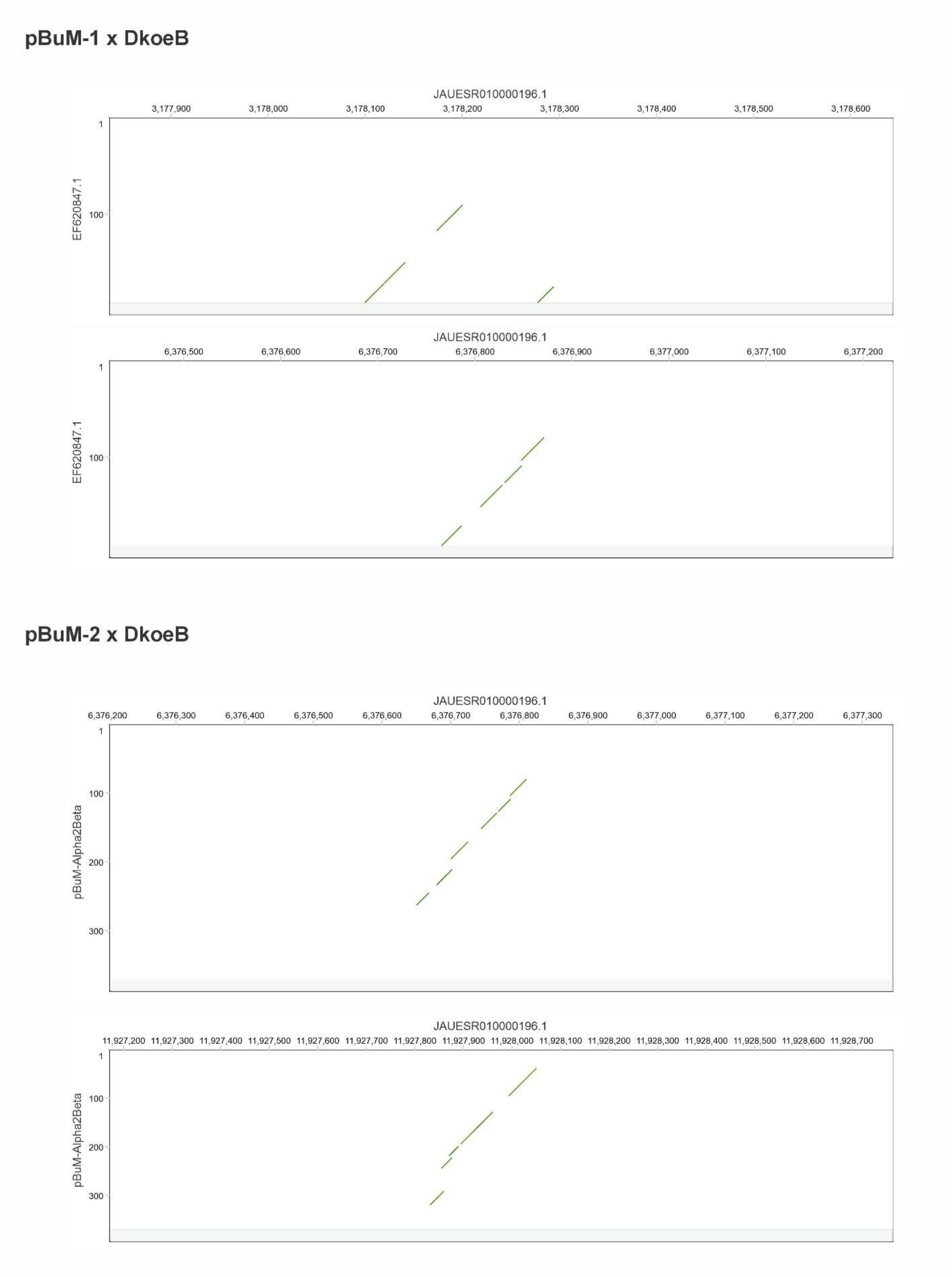

Fig. S9 Dot-plots showing the presence of pBuM in *Drosophila koepferae* Bolivian population.

```
>CDSTR138_Dkoepferae
CAACTGTTGATCTTTGTATATAGAATACGAATAAAATCA
AATATATGGTGTAGAAGAGTATATTTGCGCCGATTTACC
TTGAATATAACCTTAGTTTGATTTTTCATTCATTTATAG
TGAATATAGGCATATCTGGGC
```

Fig. S10 Dinucleotides AA/AT/TT periodicity found in the CDSTR138 consensus satDNA sequence of *D. koepferae*.

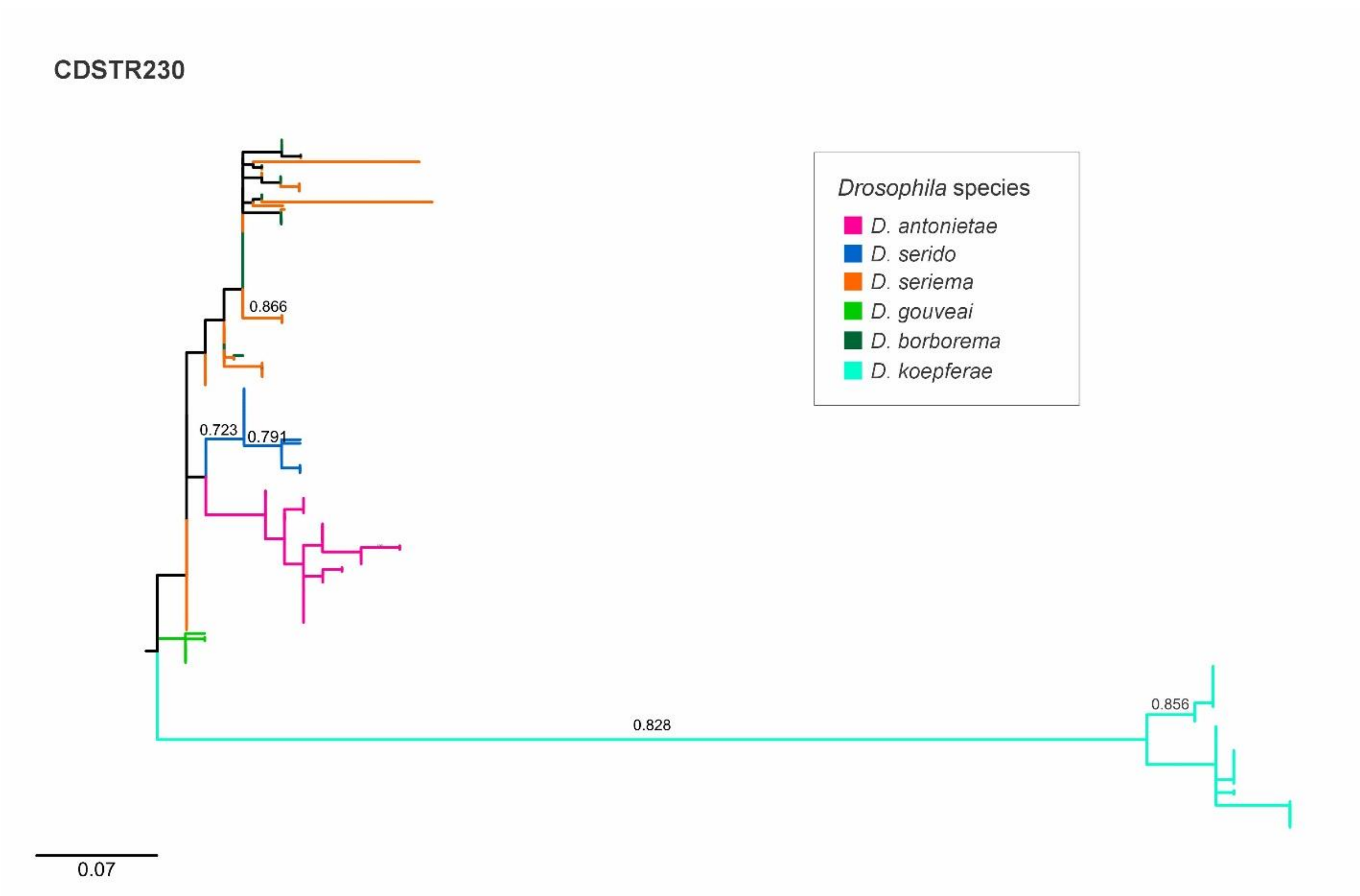

Fig. S11 Maximum-likelihood tree containing repeats of the Y-linked CDSTR230 satDNA in six species from the *buzzatii* cluster. Tree was constructed with the T92+G model, with 1000 bootstraps.
