# Supplementary material for: Satellite DNAs in Drosophila koepferae (repleta group) reveal patterns of origin, chromosomal organization, transcription, and turnover in the buzzatii cluster
Source: Chromosome Res. 2026 Sep 8;34(1):22. doi: 10.1007/s10577-026-09814-5 (PMC13553610; doi:10.1007/s10577-026-09814-5)
Supplement: Supplementary file 2 [file 10577_2026_9814_MOESM2_ESM.pdf]

Table S1. Description of all clusters retrieved from reads of Drosophila koepferae (A) by RepeatExplorer2. The satDNA families analyzed in this study are highlighted in purple.

**N input reads**	13773762
**N analyzed reads**	5904235
**N contamination reads**	5954
**N organelle reads**	50364

N nuclear reads	5847917
Nn = Na - Nc - No	

Cluster	Proportion (%)	Proportion adjusted (%)	N reads	Satellite Probability	Consensus length (pb)	C index	P index	Similarity hits	Estimated Copy Number
1	0.700	0.708	41383	1.01e-06		5.41e-01	0.0574		
2	0.600	0.601	35143	1.04e-09		3.25e-01	0.1610		
3	0.500	0.505	29505	1.21e-05		7.11e-01	0.1000		
4	0.420	0.424	24783	0.0128	616	0.702	0.506		
5	0.410	0.417	24377	9.40e-07		4.21e-01	0.6300	22.73% Class_I/LINE	
6	0.390	0.394	23023	3.59e-06		5.52e-01	0.1180		
7	0.360	0.363	21241	1.78e-05		8.22e-01	0.1650		
8	0.350	0.355	20736	0.994	40	0.993	0.968	**CDSTR8**	15956
9	0.350	0.354	20691	0.989	138	0.994	0.950	**CDSTR138**	4615
10	0.300	0.300	17534	9.29e-12		2.64e-01	0.0788		
11	0.270	0.272	15912	0.0373	225	0.985	0.734	**225TR - rDNA**	2177
12	0.270	0.269	15713	2.42e-06		9.33e-01	0.1250		
13	0.250	0.250	14618	0.0317	108	0.771	0.645	**IGS-109TR - rDNA**	4166
14	0.240	0.247	14448	0.0250	3023	0.737	0.604		147
15	0.240	0.238	13915	6.68e-12		2.54e-01	0.0977		
16	0.230	0.237	13870	1.92e-09		3.75e-01	0.0599		
17	0.220	0.220	12893	0.01460	2118	0.705	0.626	21.17% Class_I/LINE	187
18	0.220	0.219	12815	2.37e-15		1.34e-01	0.0672		
19	0.210	0.213	12455	1.95e-22		1.04e-03	0.6880	31.51% 45S_rDNA	
20	0.210	0.212	12404	0.1030	6937	0.945	0.774	6.29% Class_I/LTR/Ty3_gypsy	55
21	0.210	0.209	12226	2.16e-23		8.18e-05	0.6880	31.79% organelle/mitochondria	
22	0.200	0.202	11831	1.19e-03		6.38e-01	0.3740		
23	0.200	0.202	11823	5.53e-11		2.96e-01	0.0456		
24	0.200	0.202	11815	1.44e-23		7.72e-04	0.8340	1.90% organelle/mitochondria	
25	0.200	0.201	11774	1.39e-22		7.64e-04	0.7100	48.22% 45S_rDNA	
26	0.200	0.199	11621	0.0423	6179	0.850	0.715		58
27	0.190	0.195	11399	0.979	150	0.999	0.995	**DBC-150**	2339
28	0.190	0.195	11391	1.62e-04		8.12e-01	0.2560		
29	0.190	0.193	11268	0.0159	3840	0.711	0.534	6.61% Class_I/LTR/Bel-Pao	90
30	0.190	0.192	11243	1.12e-16		9.07e-02	0.0727		
31	0.190	0.188	10970	4.56e-08		3.75e-01	0.3340		
32	0.190	0.188	10966	0.4270	6228	0.931	0.816	6.99% Class_I/LTR/Ty3_gypsy	54
33	0.180	0.186	10890	0.00593	699	0.703	0.428		480
34	0.180	0.186	10855	3.83e-08		4.47e-01	0.0367		
35	0.180	0.177	10349	6.64e-22		3.87e-03	0.6090	27.83% Class_II/Subclass_2/Helitron	
36	0.170	0.177	10324	0.15100	5848	0.936	0.780	7.48% Class_I/LTR/Ty3_gypsy	54
37	0.170	0.168	9829	4.71e-23		1.02e-04	0.7690	13.48% organelle/mitochondria	
38	0.170	0.167	9780	8.21e-23		1.02e-04	0.7420	56.40% 45S_rDNA	
39	0.160	0.165	9632	9.15e-12		2.26e-01	0.5860	13.95% Class_II/Subclass_2/Helitron	
40	0.160	0.161	9438	2.39e-21		1.01e-02	0.6530	26.91% 45S_rDNA	
41	0.150	0.156	9103	2.09e-21		1.65e-03	0.5210	2.39% 45S_rDNA	
42	0.150	0.153	8949	1.17e-22		1.39e-03	0.7190	32.74% 45S_rDNA	
43	0.150	0.147	8576	0.5830	5449	0.938	0.827	3.09% Class_I/LTR/Ty3_gyspy	48
44	0.140	0.144	8393	1.79e-03		6.32e-01	0.7650	6.78% Class_I/LTR/Ty3_gypsy	
45	0.140	0.141	8252	0.11300	8307	0.897	0.810	6.91% Class_I/LTR/Ty3_gypsy	31
46	0.130	0.132	7724	6.61e-22		7.25e-03	0.7610	14.47% Class_I/LTR/Bel-Pao	
47	0.130	0.132	7692	1.75e-21		7.16e-03	0.6830	7.10% Class_I/LINE	
48	0.130	0.129	7550	0.0831	5317	0.906	0.784	7.80% Class_I/LTR/Ty3_gypsy	44
49	0.130	0.129	7538	0.04530	6279	0.907	0.745	9.05% Class_I/LTR/Bel-Pao	37
50	0.130	0.127	7415	1.00e-21		5.40e-04	0.5800	8.86% Class_I/LINE	
51	0.120	0.122	7126	5.67e-20		1.67e-02	0.5930	4.63% Class_I/LTR/Ty3_gypsy	
52	0.120	0.122	7107	1.39e-22		8.44e-04	0.7110	12.57% Class_I/LINE	
53	0.120	0.119	6952	0.02030	4711	0.716	0.621	5.62% Class_I/LTR/Ty3_gypsy	45
54	0.120	0.118	6907	0.733	6312	0.975	0.860	7.31% Class_I/LTR/Ty3_gypsy	34
55	0.110	0.115	6714	9.59e-19		4.69e-02	0.5800	8.21% Class_I/LINE	
56	0.110	0.111	6519	3.48e-07		4.35e-01	0.2590		
57	0.110	0.109	6363	0.2700	188	0.914	0.843	**CDSTR198**	1042
58	0.110	0.107	6274	4.27e-10		3.48e-01	0.0162		
59	0.100	0.105	6147	0.05640	4666	0.858	0.789	13.63% Class_I/LTR/Ty1_copia	41
60	0.100	0.102	5954	9.59e-01	5386	1.0000	0.999	100% contamination	34
61	0.100	0.101	5914	1.13e-07		3.68e-01	0.5720	1.69% Class_I/LTR/Ty3_gypsy	

62	0.100	0.101	5913	1.65e-22		2.54e-03	0.7000	12.06% organelle/mitochondria	
63	0.100	0.101	5878	2.70e-22		1.70e-04	0.6650	13.81% organelle/mitochondria	
64	0.098	0.099	5808	6.12e-24		1.38e-03	0.8740	23.45% Class_I/LTR/Bel-Pao	
65	0.097	0.098	5749	1.70e-16		8.19e-02	0.3890	3.15% Class_I/LINE	
66	0.097	0.098	5710	4.29e-21		2.10e-03	0.4470		
67	0.096	0.097	5666	1.51e-21		2.08e-02	0.8450	19.94% Class_II/Subclass_2/Helitron	
68	0.096	0.096	5640	7.64e-22		8.87e-04	0.6040	10.48% Class_I/LINE	
69	0.095	0.096	5638	1.13e-20		5.14e-03	0.3830		
70	0.094	0.095	5574	1.16e-14		1.24e-01	0.3990	2.08% Class_I/LINE	
71	0.094	0.095	5553	9.24e-21		8.82e-03	0.2040		
72	0.091	0.092	5353	1.66e-21		3.73e-03	0.5390		
73	0.088	0.089	5197	8.01e-07		4.21e-01	0.6500	4.56% Class_I/LTR/Bel-Pao	
74	0.088	0.089	5196	2.64e-07		3.91e-01	0.5990	3.81% Class_I/LTR/Ty3_gypsy	
75	0.085	0.086	5009	0.03010	4453	0.831	0.633	5.81% Class_I/LTR/Ty1_copia	35
76	0.084	0.085	4981	1.03e-20		1.46e-03	0.3300		
77	0.084	0.085	4951	2.70e-22		2.04e-04	0.6700		
78	0.084	0.085	4951	7.90e-03		6.68e-01	0.6340	3.96% Class_I/LTR/Ty3_gypsy	
79	0.083	0.084	4913	1.16e-20		5.70e-03	0.2950	19.99% 45S_rDNA	
80	0.083	0.083	4880	0.77500	7416	0.960	0.880	7.36% Class_I/LTR/Ty3_gypsy	20
81	0.083	0.083	4873	1.47e-19		6.36e-02	0.8200	21.05% Class_I/LTR/Bel-Pao	
82	0.080	0.080	4705	1.25e-20		2.13e-04	0.2940		
83	0.080	0.080	4703	1.53e-14		1.61e-01	0.6390	9.12% organelle/mitochondria	
84	0.078	0.079	4627	0.84600	5720	0.948	0.922	13.42% Class_I/LINE	25
85	0.077	0.077	4522	3.16e-22		1.33e-03	0.6620	20.21% Class_I/LTR/Bel-Pao	
86	0.076	0.077	4491	1.95e-22		2.90e-03	0.6900	2.03% Class_I/LTR/Ty3_gypsy	
87	0.075	0.076	4422	8.39e-20		1.61e-02	0.5490		
88	0.073	0.074	4310	4.46e-18		6.38e-02	0.5500		
89	0.073	0.073	4283	4.75e-18		8.86e-02	0.7860	7.75% Class_I/LTR/Ty3_gypsy	
90	0.069	0.069	4064	0.976	261	0.991	0.938	**CDSTR230**	479
91	0.068	0.069	4038	3.63e-20		2.08e-02	0.6270	3.27% Class_I/LTR/Ty3_gypsy	
92	0.068	0.068	4000	0.04530	6115	0.896	0.744	10.38% Class_I/LTR/Bel-Pao	20
93	0.068	0.068	3997	2.43e-14		1.54e-01	0.5570		
94	0.067	0.067	3937	1.66e-21		2.79e-03	0.5410	6.02% Class_I/LTr/Bel-Pao	
95	0.063	0.064	3724	2.91e-21		1.88e-03	0.4930		
96	0.063	0.064	3715	2.25e-19		2.29e-02	0.4120		
97	0.063	0.064	3715	3.52e-03		6.48e-01	0.7200	10.34% Class_I/LTR/Ty1_copia	
98	0.062	0.063	3661	7.64e-22		2.73e-04	0.6010		
99	0.062	0.062	3635	4.69e-21		2.75e-03	0.4440		
100	0.061	0.062	3630	0.0141	958	0.709	0.739	3.25% Class_I/LTR/Ty3_gypsy	117
101	0.061	0.062	3600	2.16e-17		7.44e-02	0.5250	1.25% Class_I/LTR/Ty3_gypsy	
102	0.059	0.060	3504	0.06260	3593	0.856	0.816		30
103	0.059	0.060	3491	1.54e-18		5.29e-02	0.5170		
104	0.059	0.059	3474	2.09e-15		1.20e-01	0.5710		
105	0.059	0.059	3466	3.06e-04		5.65e-01	0.6440	6.61% Class_I/LTR/Ty3_gypsy	
106	0.058	0.059	3443	0.01400	4247	0.710	0.758	12.31% Class_I/LTR/Ty1_copia	25
107	0.055	0.055	3231	0.0458	4838	0.842	0.727	6.87% Class_I/LTR/Ty3_gypsy	21
108	0.053	0.054	3135	7.61e-24		3.19e-04	0.8560	6.00% Class_I/LINE	
109	0.053	0.053	3127	2.61e-21		4.35e-03	0.5040		
110	0.053	0.053	3127	1.09e-16		9.15e-02	0.5980	10.11% Class_I/LINE	
111	0.052	0.052	3059	0.6600	5375	0.940	0.888		18
112	0.052	0.052	3045	6.30e-21		5.29e-03	0.5260		
113	0.051	0.037	2144	0.0651	177	0.843	0.844		373
114	0.049	0.050	2902	9.52e-03		6.81e-01	0.6430		
115	0.049	0.050	2895	4.29e-22		3.45e-03	0.6360		
116	0.049	0.049	2874	3.93e-24		3.48e-04	0.8860	10.47% Class_I/LTR/Ty3_gypsy	
117	0.048	0.049	2845	0.05380	4936	0.837	0.783	6.96% Class_I/LTR/Ty3_gypsy	18
118	0.046	0.047	2725	2.99e-10		2.73e-01	0.5430		
119	0.046	0.046	2698	4.98e-22		2.97e-03	0.6310	10.86% Class_I/LINE	
120	0.046	0.046	2695	0.979	153	0.997	0.993		542
121	0.046	0.046	2692	6.47e-05		5.33e-01	0.7250		
122	0.045	0.046	2685	8.21e-23		3.72e-04	0.7370	13.18% Class_I/LTR/Ty3_gypsy	
123	0.045	0.046	2680	6.84e-23		1.49e-03	0.7500	25.90% Class_I/LINE	
124	0.045	0.045	2651	2.90e-21		4.15e-03	0.4940		
125	0.045	0.045	2637	6.81e-07		4.30e-01	0.7410		
126	0.044	0.045	2622	2.90e-21		1.49e-02	0.6300	8.96% Class_I/LINE	
127	0.044	0.045	2621	5.12e-21		1.92e-03	0.4280		
128	0.044	0.045	2606	7.49e-21		6.41e-03	0.4970	6.41% Class_I/LINE	
129	0.044	0.044	2592	3.21e-21		2.38e-03	0.4750		
130	0.044	0.044	2582	3.91e-21		3.88e-03	0.4620		
131	0.042	0.042	2455	7.35e-03		6.75e-01	0.8320	1.83% Class_I/LINE	
132	0.041	0.041	2392	3.91e-21		4.75e-03	0.4640		
133	0.040	0.041	2389	3.91e-23		5.02e-03	0.9420	13.35% Class_I/LINE	

134	0.040	0.041	2383	3.21e-21		2.97e-03	0.4790		
135	0.040	0.041	2369	6.33e-19		4.47e-02	0.4940	2.45% Class_I/LINE	
136	0.040	0.040	2364	2.61e-21		3.44e-03	0.4950		
137	0.040	0.040	2353	4.69e-21		3.01e-03	0.4410		
138	0.039	0.040	2314	4.19e-20		3.89e-02	0.7490		
139	0.039	0.039	2309	3.44e-19		1.69e-02	0.2130		
140	0.038	0.039	2267	5.12e-21		1.33e-03	0.4310		
141	0.037	0.037	2189	3.07e-16		1.38e-01	0.8120	7.81% Class_I/LTR/Ty3_gypsy	
142	0.037	0.037	2167	1.55e-03		5.99e-01	0.5580	3.00% Class_I/LTR/Ty3_gypsy	
143	0.036	0.037	2144	0.0651	177	0.843	0.844	**CDSTR177**	373
144	0.035	0.035	2076	8.21e-23		1.93e-03	0.7390		
145	0.035	0.035	2044	2.48e-18		4.79e-02	0.4310		
146	0.034	0.034	1996	1.14e-20		5.01e-04	0.3100		
147	0.032	0.033	1915	7.64e-22		2.09e-03	0.5980		
148	0.032	0.032	1889	1.81e-05		4.91e-01	0.6640	5.35% Class_I/LTR/Ty1_copia	
149	0.032	0.032	1886	1.92e-15		9.60e-02	0.4630		
150	0.032	0.032	1862	9.97e-18		6.12e-02	0.3700		
151	0.031	0.032	1847	2.59e-10		2.69e-01	0.5590	6.98% Class_I/LTR/Ty3_gypsy	
152	0.031	0.031	1830	8.58e-22		9.84e-03	0.7380	5.74% Class_I/LTR/Ty3_gypsy	
153	0.031	0.031	1803	2.09e-21		1.66e-03	0.5230	41.93% Class_II/Subclass_2/Helitron	
154	0.030	0.031	1800	7.34e-21		1.39e-02	0.1490		
155	0.030	0.030	1766	2.13e-19		4.36e-02	0.6190	1.53% Class_I/LINE	
156	0.029	0.029	1725	3.78e-21		7.17e-03	0.6030		
157	0.028	0.028	1638	0.73300	4231	0.982	0.855	15.02% Class_I/LTR/Ty1_copia	12
158	0.028	0.028	1632	0.03860	4119	0.824	0.684	10.66% Class_I/LTR/Ty1_copia	12
159	0.028	0.028	1629	0.846	450	0.952	0.921		111
160	0.027	0.027	1603	1.66e-21		3.75e-03	0.5410	12.79% Class_I/LINE	
161	0.027	0.027	1575	1.98e-19		2.52e-02	0.5320		
162	0.026	0.027	1572	2.39e-21		5.09e-03	0.6550	2.74% Class_I/LTR/Ty3_gypsy	
163	0.026	0.027	1562	5.12e-21		2.63e-03	0.4320		
164	0.026	0.026	1546	1.20e-20		6.47e-04	0.2990		
165	0.026	0.026	1530	4.29e-21		6.70e-04	0.4500		
166	0.025	0.026	1500	2.70e-22		2.67e-03	0.6720	9.53% Class_I/LTR/Ty3_gypsy	
167	0.025	0.025	1465	2.61e-21		6.83e-04	0.5030	10.31% Cass_I/LINE	
168	0.024	0.024	1407	1.95e-22		2.13e-03	0.6870	10.23% Class_I/LTR/Bel-Pao	
169	0.023	0.023	1374	0.50900	4349	0.937	0.822	13.90% Class_I/LTR/Ty1_copia	10
170	0.023	0.023	1369	1.29e-21		7.35e-04	0.5630	1.53% Class_I/LTR/Ty1_copia	
171	0.022	0.022	1297	1.54e-16		9.18e-02	0.5760		
172	0.022	0.022	1296	8.50e-21		9.29e-03	0.1790		
173	0.022	0.022	1286	2.90e-21		2.33e-03	0.4940		
174	0.022	0.022	1271	3.55e-21		2.36e-03	0.4740		
175	0.021	0.021	1225	2.34e-21		2.48e-03	0.5070		
176	0.021	0.021	1215	8.64e-21		3.29e-03	0.3580	3.29% Class_II/Subclass_2/Helitron	
177	0.019	0.020	1146	1.09e-20		5.24e-03	0.4040		
178	0.019	0.020	1141	1.17e-22		8.78e-04	0.7180	7.36% Class_I/LTR/Ty3_gypsy	
179	0.019	0.019	1133	9.75e-21		3.58e-03	0.3420		
180	0.018	0.019	1088	0.05180	4221	0.962	0.749	14.25% Class_I/LTR/Ty1_copia	8
181	0.017	0.017	1018	3.91e-21		2.95e-03	0.4580	13.65% Class_I/LINE	
182	0.017	0.017	995	1.12e-20		2.41e-02	0.7210	10.45% Class_I/LTR/Bel-Pao	
183	0.016	0.016	925	1.31e-03		6.15e-01	0.8500		
184	0.015	0.015	886	4.29e-22		1.16e-03	0.6410		
185	0.015	0.015	886	0.981	1979	0.975	0.960		14
186	0.015	0.015	885	5.16e-14		1.53e-01	0.5050		
187	0.015	0.015	857	0.742	235	0.937	0.930		112
188	0.014	0.014	841	2.70e-22		1.19e-03	0.6720	28.89% Class_I/LTR/Ty3_gypsy	
189	0.014	0.014	831	1.58e-20		1.20e-03	0.1150		
190	0.013	0.013	770	1.53e-20		1.30e-03	0.0969		
191	0.013	0.013	756	3.90e-23		1.32e-03	0.7830	22.88% Class_I/LTR/Ty3_gypsy	
192	0.013	0.013	754	1.66e-21		1.33e-03	0.5420	16.18% Class_I/LTR/Ty3_gypsy	
193	0.013	0.013	753	4.71e-23		1.33e-03	0.7720		
194	0.013	0.013	750	1.95e-22		1.36e-03	0.6890		
195	0.013	0.013	750	1.59e-20		1.33e-03	0.2040		
196	0.013	0.013	748	2.61e-21		1.37e-03	0.5020	2.27% Class_I/LTR/Ty1_copia	
197	0.012	0.012	728	1.14e-20		4.12e-03	0.3120		
198	0.012	0.012	703	1.11e-20		8.53e-03	0.3870		
199	0.011	0.011	656	1.44e-23		1.52e-03	0.8320		
200	0.011	0.011	627	4.71e-23		1.59e-03	0.7710	19.30% Class_I/LINE	
201	0.010	0.010	596	3.16e-22		1.68e-03	0.6560	9.06% Class_I/LTR/Ty3_gypsy	

Table S2. Description of all clusters retrieved from reads of Drosophila koepferae (B) by RepeatExplorer2. The satDNA families analyzed in this study are highlighted in purple.

**N input reads**	69558804	N nuclear reads	6270997
**N analyzed reads**	6394359	Nn = Na - Nc - No	
**N contamination reads**	6043		
**N organelle reads**	117319		

Cluster	Proportion (%)	Proportion adjusted (%)	N reads	Satellite Probability	Consensus length (pb)	C index	P index	Similarity hits	Estimated Copy Number
1	0.650	0.661	41466	3.69e-06		7.01e-01	0.0464		
2	0.540	0.546	34243	3.05e-14		1.58e-01	0.1220		
3	0.380	0.383	24049	0.986	40	0.995	0.973	**CDSTR8**	17257
4	0.370	0.376	23567	5.35e-14		1.73e-01	0.1090		
5	0.360	0.369	23146	0.01200	558	0.712	0.495		1191
6	0.360	0.362	22728	1.31e-11		2.44e-01	0.6330	23.94% Class_I/LINE	
7	0.320	0.324	20301	7.10e-06		8.56e-01	0.1500		
8	0.310	0.314	19711	2.10e-06		7.17e-01	0.0402		
9	0.270	0.279	17489	0.989	138	0.992	0.951	**CDSTR138**	3638
10	0.270	0.276	17328	1.65e-22		6.93e-04	0.6980	20.35% organelle/mitochondria	
11	0.270	0.273	17096	1.79e-06		7.89e-01	0.0706		
12	0.270	0.271	16970	5.04e-13		2.05e-01	0.1370		
13	0.260	0.265	16627	9.10e-19		3.62e-01	0.0387		
14	0.260	0.263	16483	4.33e-10		3.27e-01	0.0922		
15	0.250	0.260	16276	0.04940	108	0.986	0.765	**IGS-109TR - rDNA**	4326
16	0.240	0.246	15406	3.16e-22		5.95e-04	0.6620		
17	0.210	0.215	13484	6.64e-22		1.04e-03	0.6150	4.29% organelle/mitochondria	
18	0.210	0.213	13351	7.46e-07		9.42e-01	0.0926		
19	0.210	0.212	13303	0.02140	2064	0.807	0.567		185
20	0.200	0.201	12633	3.07e-08		4.43e-01	0.0501		
21	0.200	0.201	12624	0.891	6306	0.983	0.903	7.05% Class_I/LTR/Ty3_gypsy	57
22	0.190	0.198	12442	3.47e-21		9.49e-03	0.6070	19.23% Class_I/LINE	
23	0.190	0.196	12262	3.87e-13		2.13e-01	0.0621		
24	0.190	0.189	11858	4.83e-11		2.85e-01	0.0762		
25	0.180	0.189	11823	8.75e-22		3.43e-04	0.5950	20.21% organelle/mitochondria	
26	0.180	0.187	11723	5.76e-22		5.12e-04	0.6180	55.11% organelle/mitochondria	
27	0.180	0.181	11344	1.00e-21		9.17e-04	0.5770	9.30% organelle/mitochondria	
28	0.180	0.180	11269	1.00e-21		7.99e-04	0.5790	21.41% organelle/mitochondria	
29	0.170	0.176	11036	4.57e-20		1.54e-02	0.6100	27.44% Class_II/Subclass_2/Helitron	
30	0.170	0.175	10987	4.38e-22		8.19e-03	0.7910	8.36% Class_I/LTR/Bel-Pao	
31	0.170	0.173	10829	1.82e-11		2.40e-01	0.5960	14.35% Class_II/Subclass_2/Helitron	
32	0.170	0.172	10795	4.26e-03		6.90e-01	0.4080		
33	0.170	0.171	10736	0.01510	3839	0.717	0.512	6.65% Class_I/LTR/Bel-Pao	80
34	0.170	0.170	10660	0.05990	5449	0.851	0.810	3.60% Class_I/LTR/Ty3_gypsy	56
35	0.170	0.169	10597	1.17e-22		5.66e-04	0.7190	45.56% 45S_rDNA	
36	0.160	0.166	10416	1.29e-21		9.61e-05	0.5570		
37	0.160	0.163	10241	0.02680	225	0.962	0.670	**225TR - rDNA**	1306
38	0.150	0.154	9649	1.14e-14		1.58e-01	0.0277		
39	0.150	0.152	9524	9.81e-23		1.05e-04	0.7280	46.17% 45S_rDNA	
40	0.150	0.150	9381	3.92e-17		7.36e-02	0.3170		
41	0.150	0.148	9274	0.17400	6147	0.914	0.812	6.06% Class_I/LTR/Ty3_gypsy	43
42	0.140	0.147	9207	4.19e-12		2.21e-01	0.2340		
43	0.140	0.144	9058	6.64e-22		6.62e-04	0.6080	24.39% 45S_rDNA	
44	0.140	0.144	9018	4.22e-15		1.07e-01	0.3330		
45	0.140	0.143	8998	9.74e-22		1.39e-02	0.7320	12.67% Class_I/LINE	
46	0.140	0.142	8909	4.69e-21		5.61e-04	0.4360	7.45% organelle/plastid	
47	0.140	0.141	8847	3.55e-21		1.13e-04	0.4730	10.40% organelle/mitochondria	
48	0.140	0.140	8788	1.87e-10		2.65e-01	0.4340	10.16% organelle/mitochondria	
49	0.140	0.140	8778	3.16e-22		1.03e-03	0.6560	33.53% 45S_rDNA	
50	0.140	0.140	8766	3.81e-06		4.68e-01	0.7770	6.79% Class_I/LTR/Ty3_gypsy	
51	0.130	0.137	8593	0.05800	5848	0.852	0.801	5.03% Class_I/LTR/Ty3_gypsy	42
52	0.130	0.137	8587	3.80e-22		6.87e-03	0.7980	8.82% Class_I/LT3/Ty3_gypsy	
53	0.130	0.134	8412	5.53e-20		4.35e-02	0.7270	7.94% Class_I/LTR/Ty3_gypsy	
54	0.130	0.134	8377	2.90e-21		1.19e-04	0.4850		
55	0.130	0.134	8375	1.34e-06		4.22e-01	0.5850	5.29% Class_I/LTR/Ty3_gypsy	
56	0.120	0.126	7874	7.37e-17		1.09e-01	0.7180	11.85% Class_I/LTR/Bel-Pao	
57	0.120	0.121	7585	1.76e-23		4.61e-03	0.8160	7.70% Class_I/LTR/Ty3_gypsy	
58	0.120	0.120	7521	6.04e-21		1.33e-04	0.4120	5.90% organelle/mitochondria	
59	0.120	0.120	7499	1.86e-21		1.20e-03	0.5320	6.69% Class_I/LINE	
60	0.120	0.118	7403	6.53e-21		1.35e-04	0.3970		
61	0.120	0.117	7355	1.95e-21		9.25e-03	0.6740	9.04% Class_I/LINE	
62	0.110	0.116	7292	1.04e-02		6.94e-01	0.6760	9.35% Class_I/LTR/Ty3_gypsy	

63	0.110	0.115	7242	1.95e-22		3.14e-03	0.6850	28.14% 45S_rDNA	
64	0.110	0.111	6968	8.45e-20		3.14e-02	0.6180	4.74% Class_I/LTR/Ty3_gypsy	
65	0.110	0.109	6830	5.69e-23		2.49e-03	0.7580	7.23% Class_I/LINE	
66	0.110	0.108	6758	2.82e-18		4.69e-02	0.1800		
67	0.100	0.104	6552	3.84e-08		4.10e-01	0.1460	1.28% Class_I/LTR/Ty1_copia	
68	0.100	0.104	6500	4.69e-21		1.54e-03	0.4350		
69	0.100	0.102	6399	1.95e-22		4.07e-03	0.6920	8.97% Class_I/LINE	
70	0.097	0.099	6234	0.9010	7420	0.969	0.898	7.88% Class_I/LTR/Ty3_gypsy	24
71	0.096	0.098	6141	0.979	150	0.999	0.992	**DBC-150**	1175
72	0.096	0.098	6127	0.0378	6279	0.914	0.714	10.48% Class_I/LTR/Bel-Pao	28
73	0.095	0.096	6043	9.59e-01	5386	1.00e+00	1,000	100% contamination	32
74	0.094	0.096	6040	1.15e-20		7.95e-03	0.3740		
75	0.093	0.094	5923	1.30e-20		3.78e-02	0.8260	19.30% Class_II/Subclass_2/Helitron	
76	0.092	0.094	5893	0.42700	187	0.931	0.816	**CDSTR198**	905
77	0.092	0.094	5872	0.03190	3525	0.783	0.627	5.01% Class_I/LTR/Ty1_copia	48
78	0.091	0.093	5817	3.21e-21		1.94e-03	0.4840	1.87% 45S_rDNA	
79	0.090	0.092	5782	1.17e-23		8.65e-04	0.8360	20.98% Class_I/LINE	
80	0.090	0.092	5759	1.14e-20		2.26e-03	0.3150	15.63% organelle/mitochondria	
81	0.086	0.088	5518	7.18e-05		5.27e-01	0.6980	4.98% Class_I/LTR/Ty3_gypsy	
82	0.085	0.086	5415	4.22e-08		3.64e-01	0.3850		
83	0.084	0.085	5340	1.20e-20		1.87e-04	0.2960		
84	0.082	0.083	5235	1.48e-20		1.91e-04	0.2380	17.40% organelle/mitochondria	
85	0.081	0.082	5169	1.17e-23		7.74e-04	0.8450	23.37% Class_I/LTR/Bel-Pao	
86	0.081	0.082	5161	1.29e-21		1.55e-03	0.5630	14.01% Class_I/LINE	
87	0.080	0.082	5126	0.0434	2647	0.885	0.725	3.45% Class_I/LTR/Ty3_gypsy	56
88	0.079	0.081	5066	6.25e-03		6.63e-01	0.6330	6.69% Class_I/LTR/Bel-Pao	
89	0.078	0.080	5009	3.93e-06		4.44e-01	0.5010	3.37% Class_I/LTR/Ty3_gypsy	
90	0.077	0.078	4895	1.80e-15		9.66e-02	0.4680	5.74% Class_I/LINE	
91	0.076	0.078	4865	3.21e-21		2.48e-03	0.4800		
92	0.075	0.076	4773	2.25e-08		3.68e-01	0.7810		
93	0.074	0.076	4744	8.95e-09		3.31e-01	0.6140		
94	0.074	0.076	4739	0.0544	4667	0.857	0.778	14.81% Class_I/LTR/Ty1_copia	29
95	0.073	0.075	4677	7.49e-21		1.07e-02	0.5000		
96	0.073	0.074	4659	1.57e-20		4.29e-03	0.2130		
97	0.072	0.074	4617	0.05170	4861	0.816	0.778	2.49% Class_I/LTR/Ty3_gypsy	27
98	0.072	0.074	4611	2.31e-19		2.75e-02	0.5060		
99	0.072	0.073	4606	3.15e-04		5.68e-01	0.7050	2.84% Class_I/LTR/Ty3_gypsy	
100	0.071	0.072	4535	8-74e-19		4.09e-02	0.4270		
101	0.070	0.072	4507	1.62e-20		2.82e-03	0.1760		
102	0.069	0.071	4439	1.44e-23		6.76e-04	0.8270	3.56% Class_I/LINE	
103	0.069	0.067	4199	1.25e-20		2.62e-03	0.2940		
104	0.066	0.067	4198	7.61e-24		2.38e-04	0.8640	29.35% organelle/mitochondria	
105	0.065	0.066	4148	0.02540	4836	0.743	0.641	7.16% Class_I/LTR/Ty3_gypsy	25
106	0.065	0.066	4145	0.9750	3598	0.979	0.945		33
107	0.065	0.066	4129	3.47e-21		1.31e-02	0.6100		
108	0.064	0.065	4078	0.0276	6107	0.753	0.727	9.44% Class_I/LTR/Bel-Pao	19
109	0.063	0.064	4011	2.34e-21		2.00e-03	0.5050	10.12% Class_I/LINE	
110	0.060	0.061	3816	3.29e-18		4.63e-02	0.2930		
111	0.059	0.060	3790	5.12e-15		1.06e-01	0.3820		
112	0.059	0.060	3776	1.42e-16		7.54e-02	0.4680		
113	0.058	0.060	3737	0.02040	861	0.731	0.739		125
114	0.058	0.060	3734	3.43e-20		4.98e-02	0.8450		
115	0.057	0.058	3630	9.67e-03		6.86e-01	0.7130	4.27% Class_I/LTR/Ty3_gypsy	
116	0.056	0.057	3589	1.11e-20		9.19e-03	0.3920		
117	0.056	0.057	3560	1.14e-21		3.65e-03	0.5710	5.31% Class_I/LINE	
118	0.056	0.057	3559	1.60e-03		6.18e-01	0.6960	9.08% Class_I/LTR/Ty1_copia	
119	0.055	0.056	3537	9.21e-03		6.84e-01	0.6460		
120	0.055	0.056	3533	2.90e-21		4.58e-03	0.4920		
121	0.051	0.052	3278	9.72e-21		9.24e-03	0.4420	3.72% Class_I/LTR/Ty1_copia	
122	0.051	0.052	3241	2.37e-19		3.58e-02	0.6060	3.21% Class_I/LTR/Ty3_gypsy	
123	0.051	0.052	3236	2.31e-19		3.12e-02	0.5050		
124	0.050	0.051	3185	7.49e-21		5.48e-03	0.5020		
125	0.050	0.051	3177	1.17e-22		1.26e-03	0.7240	14.20% Class_I/LTR/Ty3_gypsy	
126	0.048	0.049	3079	1.92e-15		9.76e-02	0.4630		
127	0.047	0.048	3015	1.25e-16		9.52e-02	0.6360		
128	0.047	0.048	3007	5.55e-18		6.39e-02	0.5170		
129	0.047	0.048	2991	0.8830	3100	0.930	0.951	1.00% Class_I/LINE	28
130	0.046	0.047	2963	5.12e-21		4.07e-03	0.4310	1.86% Class_I/LTR/Bel-Pao	
131	0.045	0.046	2897	1.05e-20		6.90e-03	0.2400		
132	0.044	0.045	2840	2.90e-21		3.33e-03	0.4950		
133	0.044	0.044	2784	0.2450	4231	0.907	0.834	12.86% Class_I/LTR/Ty1_copia	19
134	0.043	0.044	2780	1.14e-21		4.68e-03	0.5660		

135	0.043	0.044	2762	9.90e-07		4.12e-01	0.5300	5.18% Class_I/LTR/Ty1_copia	
136	0.042	0.043	2667	3.06e-15		1.14e-01	0.4960		
137	0.041	0.042	2632	6.30e-21		1.33e-02	0.5310	33.47% Class_II/Subclass_2/Helitron	
138	0.040	0.041	2556	4.80e-21		1.34e-02	0.5700		
139	0.040	0.040	2527	8.09e-21		3.56e-03	0.3670		
140	0.040	0.040	2526	1.55e-07		3.84e-01	0.4190		
141	0.039	0.040	2487	0.06470	4247	0.964	0.826	15.96% Class_I/LTR/Ty1_copia	17
142	0.039	0.039	2477	8.75e-22		2.42e-03	0.5890	12.11% Class_I/LINE	
143	0.037	0.038	2366	4.69e-21		3.80e-03	0.4410		
144	0.037	0.038	2353	3.48e-19		1.78e-02	0.2320		
145	0.036	0.037	2320	3.90e-23		1.29e-03	0.7810	29.53% Class_I/LINE	
146	0.034	0.035	2202	4.29e-21		1.85e-03	0.450		
147	0.034	0.035	2201	9.69e-07		4.16e-01	0.4130		
148	0.034	0.034	2159	0.00457	622	0.774	0.421		100
149	0.034	0.034	2145	1.65e-22		2.33e-03	0.6980	7.46% Class_I/LTR/Ty3_gypsy	
150	0.033	0.034	2142	7.55e-22		1.21e-02	0.7510	7.14% Class_I/LTR/Ty3_gypsy	
151	0.033	0.034	2121	0.976	261	0.991	0.939	**CDSTR230**	233
152	0.033	0.034	2115	0.0416	4302	0.931	0.729	11.54% Class_I/LTR/Ty1_copia	14
153	0.033	0.033	2080	6.41e-12		2.05e-01	0.3870		
154	0.032	0.033	2072	1.66e-21		4.97e-03	0.5410		
155	0.032	0.032	2018	3.91e-21		1.98e-03	0.4620		
156	0.031	0.032	1996	1.03e-17		6.31e-02	0.3430		
157	0.030	0.031	1935	1.95e-22		1.55e-03	0.6900	13.95% Class_I/LINE	
158	0.030	0.031	1930	0.0494	4349	0.777	0.854	14.72% Class_I/LTR/Ty1_copia	13
159	0.030	0.030	1892	6.30e-21		6.87e-03	0.5340	11.42% Class_I/LINE	
160	0.029	0.030	1868	2.09e-21		1.65e-03	0.5220		
161	0.029	0.030	1854	0.986	123	0.995	0.989		433
162	0.029	0.029	1847	1.11e-20		5.41e-04	0.0143		
163	0.028	0.029	1810	2.09e-21		3.53e-03	0.5180		
164	0.028	0.028	1763	0.15900	547	0.949	0.784		93
165	0.028	0.028	1761	1.00e-21		2.27e-03	0.5750		
166	0.027	0.028	1754	1.14e-21		6.04e-04	0.5660		
167	0.027	0.028	1737	4.82e-18		5.76e-02	0.5370		
168	0.027	0.027	1723	0.04850	177	0.798	0.791	**CDSTR177**	279
169	0.027	0.027	1708	5.57e-21		1.76e-03	0.4170		
170	0.026	0.026	1649	1.00e-21		3.64e-03	0.5840		
171	0.026	0.026	1638	4.76e-10		2.85e-01	0.3740		
172	0.025	0.025	1593	1.39e-22		1.88e-03	0.7110	5.59% Class_I/LTR/Ty3_gypsy	
173	0.024	0.024	1527	2.15e-18		4.58e-02	0.1110	5.76% Class_I/LTR/Ty3_gypsy	
174	0.023	0.024	1476	2.29e-22		2.03e-03	0.6790		
175	0.022	0.022	1380	1.22e-20		7.25e-04	0.0345		
176	0.022	0.022	1379	3.55e-21		3.70e-03	0.4690		
177	0.021	0.022	1369	1.10e-21		5.84e-03	0.7220	8.18% Class_I/LTR/Ty3_gypsy	
178	0.021	0.022	1361	0.05740	54	0.841	0.796	**CDSTR54**	723
179	0.021	0.022	1355	3.91e-21		2.24e-03	0.4570		
180	0.021	0.022	1350	4.02e-21		9.63e-03	0.0580		
181	0.021	0.021	1335	6.04e-21		2.29e-03	0.4130		
182	0.020	0.021	1286	5.12e-06		4.75e-01	0.7190		
183	0.020	0.020	1254	3.91e-21		3.99e-03	0.4560	15.63% Class_I/LINE	
184	0.018	0.018	1136	0.43500	152	0.918	0.906		215
185	0.017	0.017	1097	0.1520	4220	0.956	0.775	14.77% Class_I/LTR/Ty1_copia	7
186	0.017	0.017	1094	7.49e-21		1.37e-02	0.4990		
187	0.017	0.017	1086	1.60e-20		9.21e-04	0.1300		
188	0.016	0.017	1049	9.66e-25		9.53e-04	0.9460		
189	0.016	0.016	1023	6.12e-24		9.78e-04	0.8670	11.93% Class_I/LTR/Bel-Pao	
190	0.016	0.016	1002	5.32e-15		1.11e-01	0.3970		
191	0.015	0.015	963	7.04e-21		1.04e-03	0.3920		
192	0.015	0.015	958	6.70e-21		5.22e-03	0.5160	5.85% Class_I/LTR/Ty3_gypsy	
193	0.014	0.015	926	1.47e-21		1.13e-03	0.5460	1.51% Class_I/LTR/Ty1_copia	
194	0.014	0.014	905	0.07970	450	0.875	0.862		58
195	0.014	0.014	879	0.985	456	0.997	0.975		55
196	0.013	0.014	862	4.98e-22		1.16e-03	0.6260	22.51% Class_I/LTR/Bel-Pao	
197	0.013	0.014	856	1.76e-23		1.17e-03	0.8210	7.83% Class_I/LTR/Ty3_gypsy	
198	0.013	0.013	835	8.09e-21		1.22e-03	0.3710		
199	0.013	0.013	830	1.63e-20		1.20e-03	0.1710		
200	0.013	0.013	823	0.979	1584	1,000	0.988		15
201	0.013	0.013	818	4.91e-24		1.22e-03	0.8850	19.44% Class_I/LINE	
202	0.013	0.013	811	3.13e-04		5.73e-01	0.9590		
203	0.012	0.012	781	1.16e-20		6.40e-03	0.3610		
204	0.011	0.012	725	3.16e-22		1.38e-03	0.6630	3.59% Class_I/LTR/Ty3_gypsy	
205	0.011	0.011	707	1.17e-22		1.41e-03	0.7200	13.15% Class_I/LTR/Ty3_gypsy	
206	0.011	0.011	676	4.27e-13		1.97e-01	0.6410	1.33% Class_I/LTR/Ty3_gypsy	

207	0.010	0.011	669	4.98e-22		1.49e-03	0.6280	35.13% Class_I/LTR/Ty3_gypsy
208	0.010	0.010	658	1.17e-22		1.52e-03	0.7180	
209	0.010	0.010	647	3.80e-22		9.27e-03	0.7970	

Table S3. The putative satellites retrieved by TAREAN for the Argentinean population of *D. koepferae*

Putative satellites (high confidence)

**Cluster**	**Proportion (%)**	**Number of reads**	**Satellite probability**	**Consensus length**	**Connected componen t index C**	**Pair component index C**	**Similarity**	**Estimated copy number**	**Reference**
8	0.350	20736	0.994	40	0.993	0.968	**CDSTR8**	78750	Rossi et al. (2025)
9	0.350	20691	0.989	138	0.994	0.950	**CDSTR138**	4565	PMID: 28659292
27	0.190	11399	0.979	150	0.999	0.995	**DBC-150**	2280	PMID: 17551842
54	0.120	6907	0.733	6312	0.975	0.860	Ty3_gypsy/LTR	34	RepeatExplorer
90	0.069	4064	0.976	261	0.991	0.938	**CDSTR230**	476	PMID: 39656291
120	0.046	2695	0.979	153	0.997	0.993	Gypsy-12_DMel-I	541	RepBase
159	0.028	1629	0.846	450	0.952	0.921	5S-rRNA	112	AJ291303
185	0.015	886	0.981	1979	0.975	0.960		14	
187	0.015	857	0.742	235	0.937	0.930		115	

Putative satellites (low confidence)

**Cluster**	**Proportion (%)**	**Number of reads**	**Satellite probability**	**Consensus length**	**Connected componen t index C**	**Pair component index C**	**Similarity**	**Estimated copy number**	**Reference**
4	0.420	24783	0.0128	616	0.702	0.506	Galileo	1227	AY756168.1
11	0.270	15912	0.0373	225	0.985	0.734	**225TR**	2160	PMID: 31856171
13	0.250	14618	0.0317	108	0.985	0.734	**IGS-109TR**	4167	PMID: 39656291
14	0.240	14448	0.0250	3023	0.737	0.604	Gypsy-34	143	RepBase local blastn
20	0.210	12404	0.1030	6937	0.945	0.774	Ty3_gypsy/LTR	54	RepeatExplorer
26	0.200	11621	0.0423	6179	0.850	0.715	Ty3_gypsy/LTR	58	RepeatExplorer
29	0.190	11268	0.0159	3840	0.711	0.534	Bel-Pao/LTR	89	RepeatExplorer
32	0.190	10966	0.4270	6228	0.931	0.816	Ty3_gypsy/LTR	55	RepeatExplorer
43	0.150	8576	0.5830	5449	0.938	0.827	Ty3_gypsy/LTR	50	RepeatExplorer
48	0.130	7550	0.0831	5317	0.906	0.784	Ty3_gypsy/LTR	44	RepeatExplorer
57	0.110	6363	0.2700	188	0.914	0.843	**CDSTR198**	1053	PMID: 28659292
100	0.061	3630	0.0141	958	0.709	0.739	Ty3_gypsy/LTR	115	RepeatExplorer
107	0.055	3231	0.0458	4838	0.842	0.727	Ty3_gypsy/LTR	20	RepeatExplorer
111	0.052	3059	0.6600	5375	0.940	0.888		17	
113	0.051	3017	0.2050	107	0.969	0.792	**CDSTR138**	858	PMID: 28659292
143	0.036	2144	0.0651	177	0.843	0.844	**CDSTR177**	366	This work

Table S4. The putative satellites retrieved by TAREAN for the Bolivian population of *D. koepferae*

Putative satellites (high confidence)

Cluster	Proportion (%)	Number of reads	Satellite probability	Consensus length	Connected component index C	Pair component index C	Similarity	Estimated copy number	Reference
3	0.380	24049	0.986	40	0.995	0.973	**CDSTR8**	85500	Rossi et al. (2025)
9	0.270	17489	0.989	138	0.992	0.951	**CDSTR138**	3522	PMID: 28659292
21	0.200	12624	0.891	6306	0.983	0.903	Ty3_gypsy/LTR	57	RepeatExplorer
71	0.096	6141	0.979	150	0.999	0.992	**DBC-150**	1152	PMID: 17551842
151	0.033	2121	0.976	261	0.991	0.939	**CDSTR230**	228	PMID: 39656291
161	0.029	1854	0.986	123	0.995	0.989	Gypsy-12_DMel-I	424	RepBase
195	0.014	878	0.985	456	0.997	0.975	Ankyrin gene	55	XM_044010202.1
200	0.013	823	0.979	1584	1.000	0.988		15	

Putative satellites (low confidence)

Cluster	Proportion (%)	Number of reads	Satellite probability	Consensus length	Connected component index C	Pair component index C	Similarity	Estimated copy number	Reference
5	0.360	23146	0.01200	558	0.712	0.495	Galileo	1161	AY756168.1
15	0.250	16276	0.04940	108	0.986	0.765	**IGS-109TR**	4167	PMID: 31856171
19	0.210	13303	0.02140	2064	0.807	0.567	Gypsy/LTR	183	RepBase local blastn
33	0.170	10736	0.01510	3839	0.717	0.512	Bel-Pao/LTR	80	RepeatExplorer
34	0.170	10660	0.05990	5449	0.851	0.81	Ty3_gypsy/LTR	56	RepeatExplorer
37	0.160	10241	0.02680	225	0.962	0.67	**225TR**	1280	PMID: 31856171
41	0.150	9274	0.17400	6147	0.914	0.812	Ty3_gypsy/LTR	44	RepeatExplorer
51	0.130	8593	0.05800	5848	0.852	0.801	Ty3_gypsy/LTR	40	RepeatExplorer
76	0.092	5893	0.42700	187	0.931	0.816	**CDSTR198**	886	PMID: 28659292
77	0.092	5872	0.03190	3525	0.783	0.627	Ty1_copia/LTR	47	RepeatExplorer
97	0.072	4617	0.05170	4861	0.816	0.778	Ty3_gypsy/LTR	27	RepeatExplorer
105	0.065	4148	0.02540	4836	0.743	0.641	Ty3_gypsy/LTR	24	RepeatExplorer
113	0.058	3737	0.02040	861	0.731	0.739	Jockey/nonLTR	121	RepBase local blastn
148	0.034	2159	0.00457	622	0.774	0.421	DNA transposon	98	RepBase local blastn
164	0.028	1763	0.15900	547	0.949	0.784		92	
168	0.027	1723	0.04850	177	0.798	0.791	**CDSTR177**	275	This work
178	0.021	1361	0.05740	54	0.841	0.796	**CDSTR54**	700	This work
184	0.018	1136	0.43500	152	0.918	0.906		213	
194	0.014	905	0.07970	450	0.875	0.862	5S-rRNA	56	AJ291301.1;AJ291302.1; AJ291303.1

Table S5. Copy number of each CDSTR8 variant identified in raw reads from two *D. koepferae* populations using 100%-identity searches (forward and reverse complement).

***Drosophila koepferae* population**	**variant A**	**variant B**	**variant C**
DkoeA	18487	93904	54222
DkoeB	102228	491099	230141
