# Supplementary material for: Satellite DNAs in Drosophila koepferae (repleta group) reveal patterns of origin, chromosomal organization, transcription, and turnover in the buzzatii cluster
Source: Chromosome Res. 2026 Sep 8;34(1):22. doi: 10.1007/s10577-026-09814-5 (PMC13553610; doi:10.1007/s10577-026-09814-5)
Supplement: Supplementary file 3 [file 10577_2026_9814_MOESM3_ESM.pdf]

Satellite DNAs in *Drosophila koepferae* (*repleta* group) reveal patterns of origin, chromosomal organization, transcription, and turnover in the *buzzatii* cluster.

Ana Mattioli Laborne, John S. Heslop-Harrison and Gustavo C.S. Kuhn

Consensus sequences of each satellite DNA found in *D. koepferae:*

```
>CDSTR138_Dkoepferae

CAACTGTTGATCTTTGTATATAGAATACGAATAAAATCAAATATATGGTGTAGAAGAGTATATT
TGCGCCGATTTACCTTGAATATAACCTTAGTTTGATTTTTCATTCATTTATAGTGAATATAGGC
ATATCTGGGC

>CDSTR230_Dkoepferae

TACTCACTTGTCTTGATTTTTTACTTCTCTTCTATTAATCTTTGTACTGACTTTCAGTGCACAT
CAGTGATGTCATGTTTTATATTAATTTTTCTAAACAGTGACGTCTAATTTATATTTAATAATAG
AATTTAAAATATCATATCTTTTACACAAATCTTATTTATACTTAGTCCTGACATTCCATATCAT
TATTTGCTCTCCAACATTCTGACATCCGCCAATATCTTATACAATATTTATGTAAGTAAATCAG
GGTTG

>DBC-150_Dkoepferae

GGTCCGGGTACTGCCCAGATCCAACACCCGCGTATAACGTTCGGCGTAGAATTTTGGGTGTCAC
CCCGTATATCAGTTGGGCCGGGGCATTGGTCAAGCCCCAGTCACATATGTATGTAGCAGGAGGC
GGCCGGGTATTGCCGAAGTCGC

>CDSTR198_Dkoepferae

GAAAATAGCCAGAAAGGTAGATTGAACATTAATGGGCAAATGGATGGATAAATAAGACTGGTCA
TCATCCAATGAACAGAATCGTGATTAAGAGATAGAAATATGATTAGAAAGTAGGATAGAAAGGT
TAGAAAGAAGGTAGAAAGGTAGTTGGTGAGATAAACCAGAAAAAAAGCTAAAAACGGCTA

>CDSTR177_Dkoepferae

AGAAATCGAACTCATCAAAGGGATCCCAAAGAAGTACAGGAACGGTATACCAAAACTAATGAGA
AGTTTTAAGCGAGTTTCGAAATACAGAGGGGTTTCATTTAGAGTTAGGAGGGTAAAAATATGAA
TATCTTAGAGATGTTCTTCACTAAAAAATCCCATTTCTTTTCCATCAAA
```
